# *GlycoMine*^*struct*^: a new bioinformatics tool for highly accurate mapping of the human N-linked and O-linked glycoproteomes by incorporating structural features

**DOI:** 10.1038/srep34595

**Published:** 2016-10-06

**Authors:** Fuyi Li, Chen Li, Jerico Revote, Yang Zhang, Geoffrey I. Webb, Jian Li, Jiangning Song, Trevor Lithgow

**Affiliations:** 1 College of Information Engineering, Northwest A&F University, Yangling 712100, China; 2 Infection and Immunity Program, Biomedicine Discovery Institute and Department of Biochemistry and Molecular Biology, Monash University, Melbourne, VIC 3800, Australia; 3 Monash Bioinformatics Platform, Monash University, Melbourne, VIC 3800, Australia; 4 Monash Centre for Data Science, Faculty of Information Technology, Monash University, Melbourne, VIC 3800, Australia; 5 Infection and Immunity Program, Biomedicine Discovery Institute and Department of Microbiology, Monash University, Melbourne, VIC 3800, Australia; 6 National Engineering Laboratory for Industrial Enzymes and Key Laboratory of Systems Microbial Biotechnology, Tianjin Institute of Industrial Biotechnology, Chinese Academy of Sciences, Tianjin 300308, China

## Abstract

Glycosylation plays an important role in cell-cell adhesion, ligand-binding and subcellular recognition. Current approaches for predicting protein glycosylation are primarily based on sequence-derived features, while little work has been done to systematically assess the importance of structural features to glycosylation prediction. Here, we propose a novel bioinformatics method called *GlycoMine*^*struct*^(http://glycomine.erc.monash.edu/Lab/GlycoMine_Struct/) for improved prediction of human N- and O-linked glycosylation sites by combining sequence and structural features in an integrated computational framework with a two-step feature-selection strategy. Experiments indicated that *GlycoMine*^*struct*^ outperformed NGlycPred, the only predictor that incorporated both sequence and structure features, achieving AUC values of 0.941 and 0.922 for N- and O-linked glycosylation, respectively, on an independent test dataset. We applied *GlycoMine*^*struct*^ to screen the human structural proteome and obtained high-confidence predictions for N- and O-linked glycosylation sites. *GlycoMine*^*struct*^ can be used as a powerful tool to expedite the discovery of glycosylation events and substrates to facilitate hypothesis-driven experimental studies.

Glycosylation is a major type of protein post-translational modification (PTM) through which a carbohydrate (i.e., a glycosyl donor) is attached to specific functional groups on target proteins (i.e., glycosyl acceptors). It is among the most complicated of PTMs occurring in protein biosynthesis^1^ and is ubiquitous across different species and cell types^1^. Glycosylation plays an important role in a myriad of biological processes involving protein folding, sorting, trafficking, degradation, and immune response^2^,^3^,^4^,^5^. Due to its fundamental importance in cell biology, protein glycosylation has also been implicated in a number of human diseases, including congenital muscular dystrophies^6^, alcoholism^7^, Alzheimer’s disease^8^, and cancer^6^.

The three major types of glycosylation, N-, O-, and C-linked glycosylation, are distinguished in the functional groups in the protein side chain being modified with the carbohydrate moiety. While little is known about the factors contributing to C-linked glycosylation, asparagine residues can be modified by N-linked glycosylation when located within a consensus sequence motif (Asn-X-Ser/Thr, where X denotes any amino acid except Pro^9^). Oligosaccharyltransferase is the central enzyme of protein N-glycosylation in eukaryotes, catalyzing the formation of an N-glycosidic linkage of oligosaccharides to the side-chain amide of target asparagine residues. This catalysis occurs selectively on consensus sequons Asn-X-Ser/Thr in substrate proteins^10^. This pathway occurs co-translationally (*i.e.* as unfolded substrate polypeptides enter the endoplasmic reticulum) or post-translationally (*i.e.* after substrate polypeptides have folded in the lumen of the endoplasmic reticulum). Since cell surface and extracellular proteins are first translocated into the endoplasmic reticulum, protein N-glycosylation is responsible for much of the glycan modification of these extracellular proteins. O-linked glycosylation involves glycan attachment to serine or threonine residues. There exists at least five classes of O-glycosyl modifications, including O-N-acetylgalactosamine (O-galNAc), O-fucose, O-glucose, O-N-acetylglucosamine (O-GlcNAc) and O-mannose^11^. These reactions can occur in the cytosol, to proteins that will remain in the cytosol or enter into the nucleus^12^,^13^, or in the *cis*-, medial- and *trans-*Golgi compartments after secretory proteins traffic from the endoplasmic reticulum^14^,^15^. To date, no biologically significant sequons have been identified for any class of O-linked glycosylation^16^. Whether it occurs in the cytosol or the Golgi compartment, O-linked glycosylation occurs post-translationally so that only some potential glycosylation sites would be available to the glycosyltransferases that mediate this PTM^11^. Likewise, only a sub-set of all Asn-X-Ser/Thr sequences will be accessed by the glycosyltransferases that catalyze N-linked glycosylation^17^. In addition to the accessibility criterion limiting O- and N-glycosylation^11^, it was suggested that sequences surrounding a potential glycosylation site and/or distances to the next glycosylation site can impact whether an acceptor Asn-X-Ser/Thr sequence is actually N-glycosylated^18^,^19^.

Mass spectrometry is perhaps now the predominant experimental method to detect protein glycosylation sites^20^,^21^. In recent years, other techniques that can perform medium and high-throughput identification and quantification of glycosylation sites (including glycan structures and glycan occupancy) have been developed and applied^22^,^23^,^24^, including flow cytometry^25^, solid-phase extraction^26^ and lectin-based methods^27^,^28^. All of these experimental approaches require considerable time and effort. This hinders their ability to keep pace with data generated from high-throughput sequencing endeavors, given the enormous volume of proteomic data generated by these and other new technologies. Compared with other important types of PTM, such as phosphorylation, acetylation, and ubiquitination, bioinformatics-based prediction of glycosylation has lagged behind^29^.

In light of this, computational approaches to address this issue are attractive options, particularly with advances in data-mining and machine-learning algorithms. Current computational tools for protein-glycosylation prediction (e.g., GlycoMine^30^, NetNGlyc^31^, NetOGlyc^32^, EnsembleGly^33^, and GPP^34^) were constructed based on sequence features, which have been widely used as a basic feature for the construction of computational models. Sequence-based features generally include physical/chemical properties (e.g., hydrophobicity and AAindex), statistical features (e.g., position-specific scoring matrices (PSSMs)), predicted features by third-party computational methods (e.g., protein secondary structure), and functional annotations from publicly available databases.

Protein structural features have not been systematically examined or incorporated for glycosylation prediction. An interplay between N-linked glycosylation sites and secondary structures was revealed, suggesting that secondary structure features are important for distinguishing glycosylation sites from non-glycosylation sites^19^,^35^. To the best of our knowledge, NGlycPred^35^ is the only tool that has incorporated protein structural features for N-linked glycosylation prediction. The balanced predictive performance of NGlycPred based on 10-fold cross-validation in terms of accuracy (ACC) was 68.7%. Furthermore, NGlycPred is limited to N-linked glycosylation-site prediction. These underline the necessity for developing an improved approach considering both sequence-derived and three-dimensional protein-structure information.

Here, we proposed a novel computational framework, *GlycoMine*^*struct*^, for N- and O-linked glycosylation-site prediction that integrates both protein-sequence and protein-structural features. This is the only computational framework to date that assembles protein-sequence and protein-structural features for both N- and O-glycosylation-site prediction. Effective feature-selection methods, combining linear support vector machine (SVM)-based feature selection and incremental feature selection, were applied to extract the most informative sequence-based and structural features for N- and O-linked glycosylation prediction. In empirical studies, our proposed method achieved outstanding predictive performance in terms of area under the curve (AUC; 0.948 and 0.923) for N- and O-linked glycosylation sites, respectively, using a benchmark dataset and outperformed NGlycPred on an independent test dataset. Additionally, we applied *GlycoMine*^*struct*^ to scan the entire human structural proteome to identify N- and O-glycosylation sites, thereby providing a comprehensive dataset to the community for further in-depth glycosylation studies and experimental investigations.

## Results

### Methodology overview

**Figure 1:**
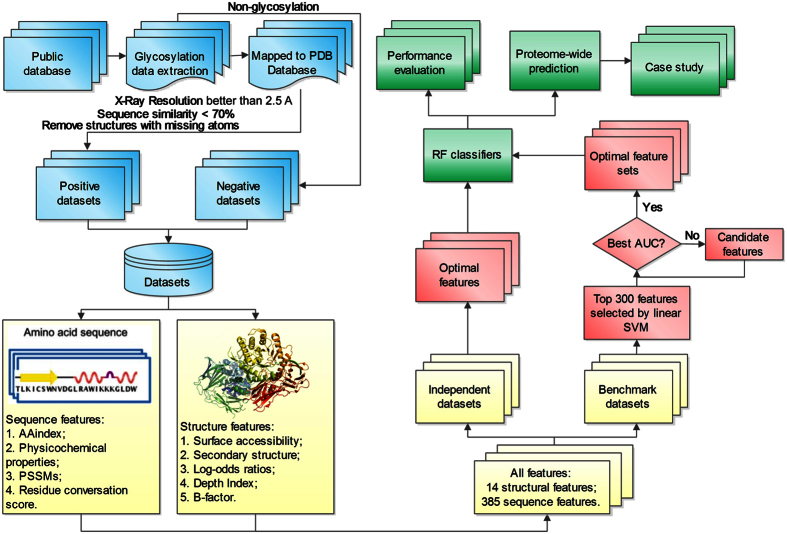
Overview of the *GlycoMine*^*struct*^ framework. Four major steps are denoted by different colors: dataset collection and preprocessing (blue), feature extraction (yellow), feature analysis and selection (red), model evaluation (green).

A flowchart describing *GlycoMine*^*struct*^ is illustrated in Fig. 1, with the four major steps denoted by different colors: dataset collection and preprocessing (blue), feature extraction (yellow), feature analysis and selection (red), and model evaluation (green). The first step involves data collection and extraction from publicly available resources. During the second step, a variety of sequence-based and structural features are extracted using third-party software. A two-step feature-selection procedure is introduced in the third step, where linear SVM-based feature selection^36^ is first used, followed by incremental feature selection (IFS)^37^ to characterize the feature subsets that contribute the most information for N- and O-linked glycosylation-site prediction. During the final stage, random forest (RF)-based classifiers are trained using the final selected optimal feature subsets (OFS) for N- and O-linked glycosylation-site prediction. The performance of RF classifiers was extensively evaluated using both cross-validation and independent tests. During this stage, we also compared the performance of our method with that of NGlycPred^35^, which is the only predictor currently integrating both sequence and structural features for N-linked glycosylation-site prediction.

### Residue enrichment of sequence motifs for both N- and O-linked glycosylation sites

**Figure 2:**
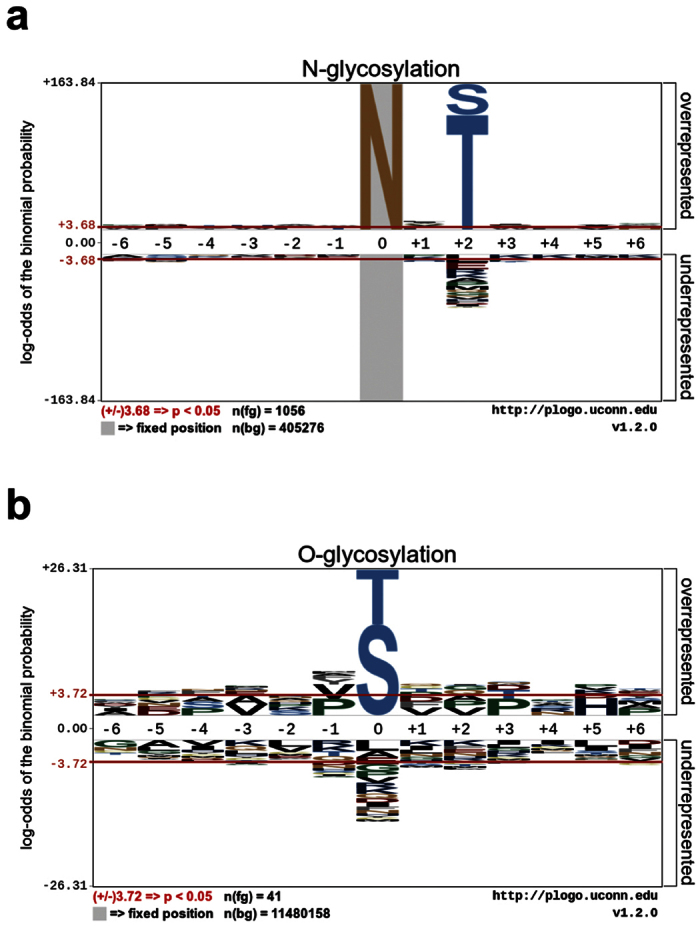
Residue specificity and enrichment of sequons. (**a**) N- and (**b**) O-linked glycosylation sites with the “human protein dataset” selected as the background set. Sequence logos and statistical test (binomial probabilities and Bonferroni correction) were generated using the pLogo program^38^.

We first analyzed the amino-acids specificity and enrichment of N- and O-linked glycosylation sites in our curated benchmark datasets. The sequons of N- and O-linked glycosylation sites were presented with a local window size of 14 residues flanking the glycosylation sites (seven residues upstream and downstream of each glycosylation site). pLogo^38^ was then applied to calculate and draw the sequence logos for N-linked (Fig. 2a) and O-linked (Fig. 2b) glycosylation sites using the human-protein dataset as background for statistical purposes. The sequence logos in Fig. 2 demonstrate the significantly overrepresented and underrepresented amino acids (*p* = 0.05) for each position of the sequons in the benchmark N- and O-linked glycosylation-site datasets.

N-linked and O-linked glycosylation sites show different preferences for neighbouring amino acids (Fig. 2). As expected, for N-linked glycosylation the central position is dominated by asparagine (N) residues; while threonine (T) and serine (S) are preferable residues at the central position of O-linked glycosylation sites. N- and O-linked glycosylation sites further showed different residue preferences at other positions. While threonine (T) and serine (S) were overrepresented in sequence motifs associated with N-glycosylation sites at position +2 (downstream of the N-glycosylation site^9^), no specific amino acids were found to be overrepresented at other O-linked glycosylation-site positions. The amino acid preferences shown in Fig. 2 represent patterns important for distinguishing N- and O-linked glycosylation sites.

### Optimized feature set (OFS)

In addition to reducing the computational complexity of classifiers, effective feature-selection methods can improve the predictive performance of classifiers by eliminating noisy and redundant features. A total of 385 sequence-derived features and 14 structural features were initially extracted using a variety of computational tools for both N- and O-glycosylation. The ‘Methods’ section and Supplementary Table S3 present a detailed description of these features. Applying the proposed two-step feature-selection method led to selection of 14 contributing features for N-linked glycosylation sites, and 11 contributing features for O-linked glycosylation sites. The IFS curves displaying the changes in AUC values during the second step of IFS are shown in Supplementary Fig. S1. The 14 N-linked and 11 O-linked optimal features were selected by five-fold cross-validation using the benchmark datasets. We also performed an independent test using these optimal features and showed that models trained using the two optimal feature sets accurately identified the N- and O-linked glycosylation sites. Tables 1 and 2 provide the lists of selected optimal features for N- and O-glycosylation, respectively. For N-linked glycosylation, the final optimal features included nine sequence-derived features and five structural features, while for O-linked glycosylation, the final optimal features included eight sequence-derived features and three structural features.

The ‘**Num**’ column in Tables 1 and 2 indicates the order of the selected features in the OFS, which were ranked by the linear SVM during the first feature selection step to quantify the importance of each individual features. The ‘**Position**’ column in Tables 1 and 2 indicates the position of a corresponding feature in the local sliding window. Refer to the subsection ‘Feature window’ of ‘Methods’ for the definition of the local sliding window. Among the selected sequence-derived features, PSSM-relevant features for different positions were chosen in the OFS for both N- and O-linked glycosylation sites. PSSM is widely used to characterize the variability of each amino acid in given protein sequences based on multiple-sequence alignment. Previous studies on predicting protein PTM sites, such as phosphorylation^39^ and ubiquitination^40^, demonstrated the importance and contribution of PSSM to prediction performance. Similarly, the feature-selection results documented in Tables 1 and 2 revealed that PSSM also plays crucial roles in predicting glycosylation sites, which is consistent with finding from our previous study^30^. Other sequence-based features were then extracted from the AAindex. A recent study showed that an appropriate degree of hydrophobicity in a glycosylation site is crucial for protein-folding mechanism, indicating a strong relationship between glycosylation and residue hydrophobicity (V1 in Table 1)^41^.

A conformational parameter involving the β-turn is another feature derived from the AAindex captured as a contributive feature for both N- and O-linked glycosylation prediction. Structural studies showed that turns and bends are regions favorable for harboring glycosylation sites as compared to other secondary structural elements^19^,^42^,^43^. Our feature selection results are consistent with these biological findings.

Tables 1 and 2 show both similarity and diversity of N- and O-linked glycosylation sites in terms of selected structural features. Absolute-accessibility features for different positions calculated by NACCESS (http://www.bioinf.manchester.ac.uk/naccess/) were selected as important features for both N- and O-linked glycosylation sites. Absolute accessibility describes a residue being conformationally accessible as a prerequisite for glycosylation^44^. Importantly, this study revealed log-odds ratios representing the epitope propensity for B cells as important attributes for N-linked glycosylation prediction. Glycosylated protein antigens play important roles in the immunologic process^45^ through binding between epitope and B cell. The log-odds ratios presented in Table 1 suggested that epitope propensity was strongly correlated with protein glycosylation. Furthermore, B factors associated with protein structural dynamics were evaluated as being contributory features for O-linked glycosylation prediction (Table 2). Given that glycosylation profoundly affects the protein folding and stability^46^, the B factor representing thermal motion and used to measure the protein stability, was revealed in our feature-selection results as strongly correlated with protein glycosylation.

Analysis of the composition of selected optimal features indicated that both sequence and structural features contributed to N- and O-linked glycosylation prediction. In the OFS of N-linked glycosylation, a total of 14 optimal features were selected, including nine sequence-derived features and five structural features. Among the nine sequence features, there were five (5/60 = 8.33%) AAindex features and four (4/360 = 1.11%) PSSM features. In the OFS of O-linked glycosylation, eight sequence features and three structural features were finally selected: the eight sequence-derived features include three AAindex (3/60 = 5%) features and five (5/300 = 1.67%) PSSM features. In comparison, sequence-derived features only accounted for 2.3% (9/385) and 2.1% (8/385) of the final selected features for N- and O-linked glycosylation, respectively, while structural features represented ~36% (5/14) and ~21% (3/14), respectively, in the final OFS. Even though the absolute number of the selected sequence-derived features was larger than that of the selected structural features, the relative percentage of the latter was larger than that of the former for both N- (~36%) and O-linked (~21%) glycosylation. This was because the number of initially extracted sequence features was much larger than that of structural features (385 *vs.* 14). Altogether, these findings suggested that structural features are indispensable and crucial for N- and O-linked glycosylation prediction.

### Feature importance and contribution in OFS

**Figure 3:**
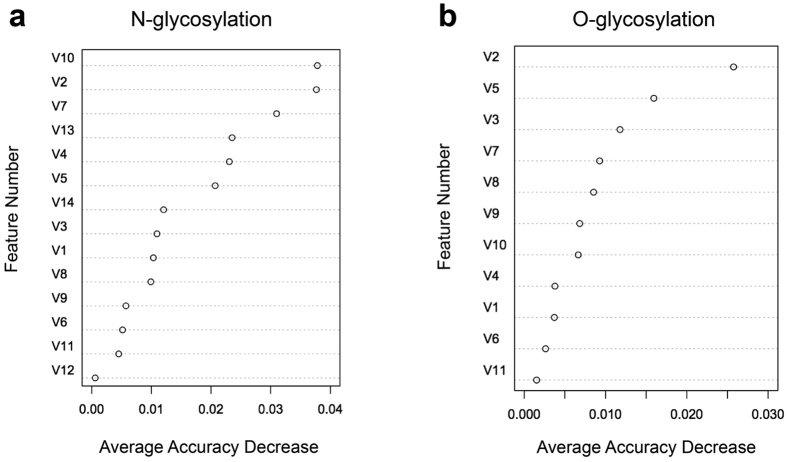
The relative importance and ranking of the selected optimal features. (**a**) N-linked glycosylation and (**b**) O-linked glycosylation based on the average accuracy decrease of models trained after removal of a correspoding feature from the feature set.

Given that the selected features in Tables 1 and 2 may or may not be equally important for glycosylation prediction, we evaluated the importance of individual optimal features in the OFS in terms of their relative contribution to the performance of N- and O-linked glycosylation prediction. Specifically, the importance of each of the features was assessed and ranked based on the average decrease in accuracy of the RF models trained using the independent test after removal of the correspoding feature from the OFS. The results are shown in Fig. 3.

#### The top two features for N-linked glycosylation-site prediction

The two most important structural features for N-linked glycosylation-site prediction (Table 1) were the log-odds ratio (V10, calculated by DiscoTope, which is for discontinuous B cell epitopes prediction) and the absolute accessibility of non-polar side chains (V2, calculated by NACCESS). Removal of each of the two features from the OFS led to the decrease in accuracy of 3.78% and 3.76%, respectively (Fig. 3a). Box plots of these two structural features between N-glycosylation and non-N-glycosylation sites (Supplementary Figs S2j and S2b) showed that N-glycosylation sites had larger average log-odds ratios (−9.596), while non-N-glycosylation sites had an average value of −11.365, suggesting the importance of glycosylation in immunological process. The difference of the average log-odds-ratio values between N-glycosylation sites and non-N-glycosylation sites was statistically significant (*p* = 0.039). Similarly, in the case of the absolute accessibility of non-polar side chains, N-glycosylation sites also had large average values of 19.504, while non-N-glycosylation sites had lower average values of 17.359, which agree with finding that non-N-glycosylation sites tend to be predicted as solvent-inaccessible. The distribution of the absolute accessibility of non-polar side chains between N-glycosylation sites and non-N-glycosylation sites was also found to be statistically significant (*p* = 0.003). The boxplots displaying differences between N-linked glycosylation and non-N-glycosylation sites for features listed in Table 1 are shown in Supplementary Fig. S2.

#### The top two features for O-linked glycosylation-site prediction

Feature importance-ranking analysis indicated that PSSM_P38 (V2, generated by BLAST) and the standard deviation of the side-chain-depth index (V5, calculated by PSAIA) were the two most important features for O-linked glycosylation-site prediction (Fig. 3b). The box plots of these two features for O-glycosylation sites and non-O-glycosylation sites are shown in Supplementary Fig. S3b,e. For the first feature, the distributions of PSSM_P38 values between O-glycosylation and non-O-glycosylation sites were significantly different, (*p* = 4.98 × 10^−8^, Supplementary Fig. S3b). The standard deviation of the side-chain-depth index is an important structural feature for O-linked glycosylation-site prediction (Table 2). The box plot in Supplementary Fig. S3e showed that the average depth-index values for O-glycosylation and non-O-glycosylation sites differed significantly (*p* = 0.003). The larger average depth-index values (0.373) for non-O-glycosylation sites relative to those of O-glycosylation sites (0.297) may be partly explained by tendency of non-O-glycosylation sites to be solvent inaccessible and buried within the protein structure. The boxplots displaying the distributions of other optimal sequence and structural feature values (Table 2) for O-linked glycosylation sites can be found in Supplementary Fig. S3.

Figure 3 was generated based on the ‘Average Accuracy Decrease’ calculated by the Random Forest algorithm after removing a certain feature from the OFS. Supplementary Figs S2 and S3, on the other hand, were drawn based on the t-test to illustrate whether the features from the OFS can significantly distinguish glycosylation sites from non-glycosylation sites (i.e., whether the distribution of the individual feature values among glycosylation sites and non-glycosylation sites was statistically different). It is important to note that these two measures are substantially different and focus on different aspects of the prediction. The t-test focuses exclusively on an individual feature’s capability of discriminating glycosylation sites from non-glycosylation sites; while the Random Forest measures the prediction accuracy decrease after removing the current features and combining the rest as the feature set for retraining the classifiers. Random Forest is a sophisticated algorithm that is capable of calculating information entropy and/or Gini index for accurately classifying the samples. Therefore, the prediction performance of Random Forest does not simply rely on the discriminatory power of an individual feature, but more so on the combination and correlation of all the available features. By way of example, although the hydrophobicity_P10 feature (V1) was capable of distinguishing glycosylation sites from non-glycosylation sites (p-value = 2.35E-43; Supplementary Fig. S2) for N-linked glycosylation, Random Forest could still achieve a better prediction performance (i.e. lower accuracy decrease; Fig. 3a) in the absence of the hydrophobicity_P10 feature (V1) by combining all other available features. Conversely, the lack of the log-ratio feature (V10; p-value = 0.039; Supplementary Fig. S2) for N-linked glycosylation resulted in a worse correlation during the model training using Random Forest and led to the largest average accuracy decrease of 0.0378 (Fig. 3a). In summary, we suggest that these two ranking schemes are both important and that they each focus on and capture different aspects of the prediction.

### Performance comparison with other tools

**Figure 4:**
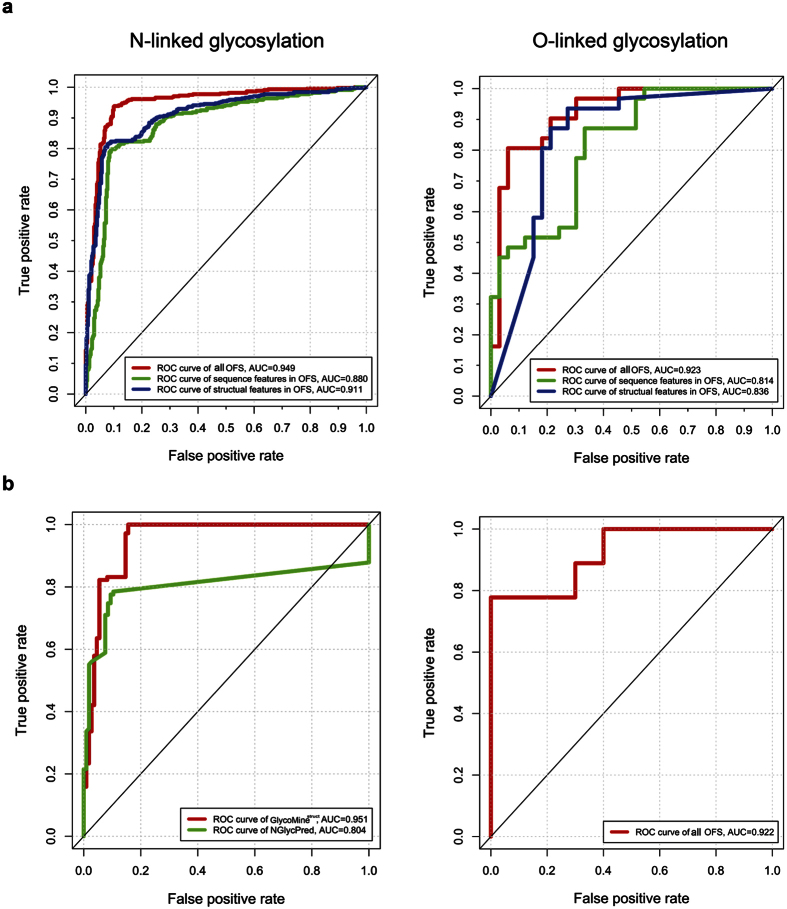
ROC curves. (**a**) Different *GlycoMine*^*struct*^ models trained with OFSs selected from all features, sequence features only, and structural features only, for N- and O-linked glycosylation sites. (**b**) N- and O-linked glycosylation-site predictions from *GlycoMine*^*struct*^ (trained with the OFS) and NGlycPred using the independent test dataset.

We evaluated and compared site-prediction performance using the OFS, only sequence features, or only structural features based on five-fold cross-validation and independent tests using the benchmark datasets. The AUC values of the models trained with different features for N- and O-glycosylation-site prediction are shown in Fig. 4a. *GlycoMine*^*struct*^ achieved the highest AUC values by combining both structural and sequence features, which suggested that both features played important roles in predicting N- and O-linked glycosylation sites.

The Receiver Operating Characteristic (ROC) curves and the corresponding AUC values showed that the models trained using the combination of sequence and structural features improved prediction of both N- and O-linked glycosylation sites as compared with models trained using only structural or sequence features. To further illustrate the predictive performance of *GlycoMine*^*struct*^, we performed an independent test using the OFS and compared the results with those from NGlycPred^35^, for N-glycosylation-site prediction. The ROC curves and AUC values of the two methods are shown in Fig. 4b. *GlycoMine*^*struct*^ outperformed NGlycPred for N-linked glycosylation-site prediction. The detailed prediction results in terms of AUC, Matthews correlation coefficient (MCC), ACC, specificity, sensitivity, and precision on both the benchmark and independent datasets are presented in Supplementary Table S1. The performance using the independent test dataset suggested that *GlycoMine*^*struct*^ outperformed NGlycPred in predicting N-linked glycosylation sites with known structural information.

### Case study

**Figure 5:**
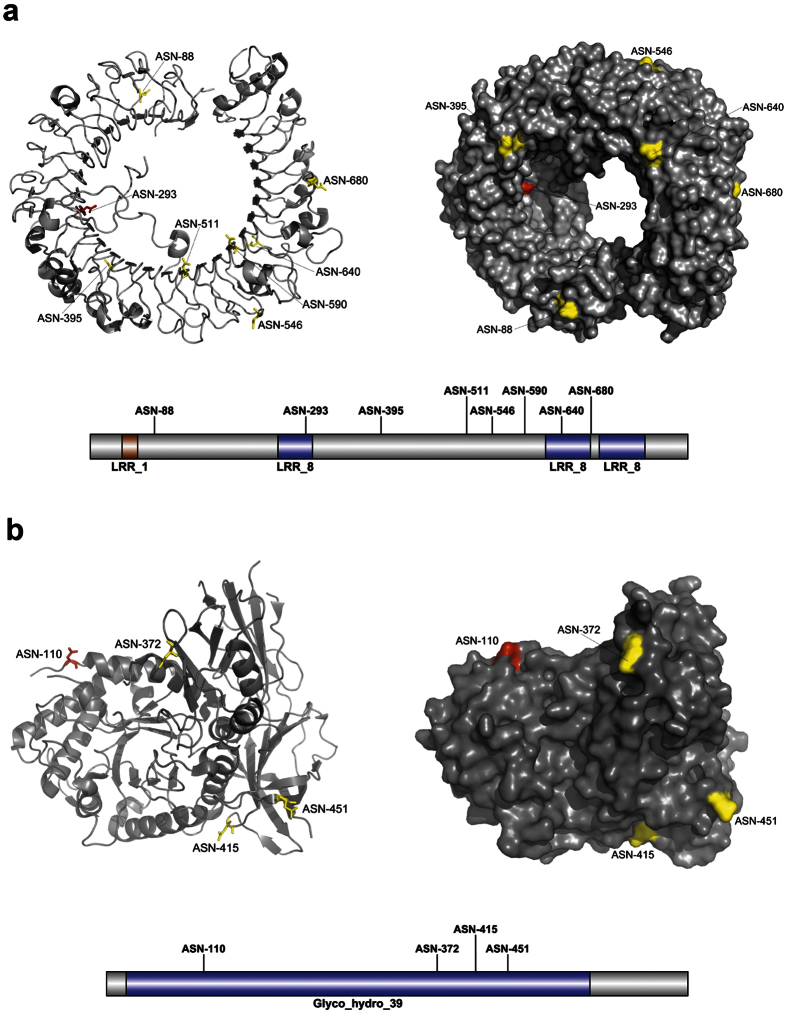
Predicted N-linked glycosylation sites from two case-study proteins using *GlycoMine*^*struct*^. (**a**) Toll-like receptor 8. (**b**) α-L-iduronidase. Predicted N-glycosylation sites from both *GlycoMine*^*struct*^ and NGlycoPred are colored in yellow, while the sites that were correctly predicted by *GlycoMine*^*struct*^, but were not predicted by NGlycPred are coloured in red. The illustrations of Pfam domains and N-glycosylation sites of these two proteins shown at the bottom of each panel were rendered using the IBS program^98^.

A case study involving the prediction of N-linked glycosylation sites in two proteins not included in the benchmark dataset illustrated the predictive capability of *GlycoMine*^*struct*^. The first protein was Toll-like receptor 8 (TLR8; PDB ID: 3WN4^47^; UniProt ID: Q9NR97; Fig. 5a), a key component of innate and adaptive immunity that controls host immune response against pathogens through recognition of molecular patterns specific to microorganisms^47^. The second protein was α-L-iduronidase (IDUA; PDB ID: 4MJ2^48^; UniProt ID: P35475; Fig. 5b), which contains a complicated structural fold consisting of a triosephosphate isomerase barrel domain harbouring the catalytic site, a β-sandwich domain, and a fibronectin-like domain, and plays an important role in hydrolyzing unsulfated alpha-L-iduronosidic linkages in dermatan sulfate^49^. Maita *et al*.^50^ noted that the crystal structure of α-L-iduronidase indicated that the protein was glycosylated on several sites, but that it contained one consensus asparagine residue in the Asn-X-Ser/Thr motif (Asn-336) that was not glycosylated. The prediction results of *GlycoMine*^*struct*^ and NGlycPred (Fig. 5) demonstrated that *GlycoMine*^*struct*^ correctly identified all experimentally verified glycosylation sites in the two proteins, while NGlycPred failed to predict glycosylation sites Asn-293^51^ of TLR8 (3WN4, chain A) and Asn-110^52^ of IDUA (4MJ2, chain A). The N-glycan attached to one predicted IDUA functional site (Asn-372) is crucial to protein function, as it enables the interaction with iduronate analogs in the active site and is required for enzymatic activity^48^. The consensus Asn-336, which is not glycosylated^50^, was predicted as such by *GlycoMine*^*struct*^. A final consensus Asn-X-Ser/Thr residue (Asn-190) that was below the prediction threshold set by *GlycoMine*^*struct*^ was shown to be subject to only partial glycosylation^50^.

### Proteome-wide prediction of N- and O-linked glycosylation substrates and sites

In order to test the capability of *GlycoMine*^*struct*^ on systems-level mapping, we performed proteome-wide glycosylation-site prediction. In order to identify novel N- and O-glycosylation substrates and sites, we downloaded and screened the human structural proteome comprising a total of 20,538 human protein structures with resolution better than 3 Å from the PDB database^53^. To obtain high-confidence prediction results, the N- and O-glycosylation models trained using the corresponding optimal features on the complete training dataset were used, with prediction thresholds adjusted to a 99% specificity level. A summary of the predicted N-linked and O-linked glycosylated substrates and glycosylation sites are shown in Supplementary Table S2. A total of 3386 and 5298 proteins were predicted to be N- and O-glycosylated substrates, respectively, containing 4996 predicted N- and 10529 O-linked glycosylation sites, respectively. As a resource for the community, these proteome-wide results can be downloaded from the *GlycoMine*^*struct*^ website, enabling users to obtain the proteome-wide N- and O-glycosylation-site prediction results for their experimental verification.

### Functional enrichment analysis of predicted N- and O-linked glycosylated proteins at the proteome level

**Figure 6:**
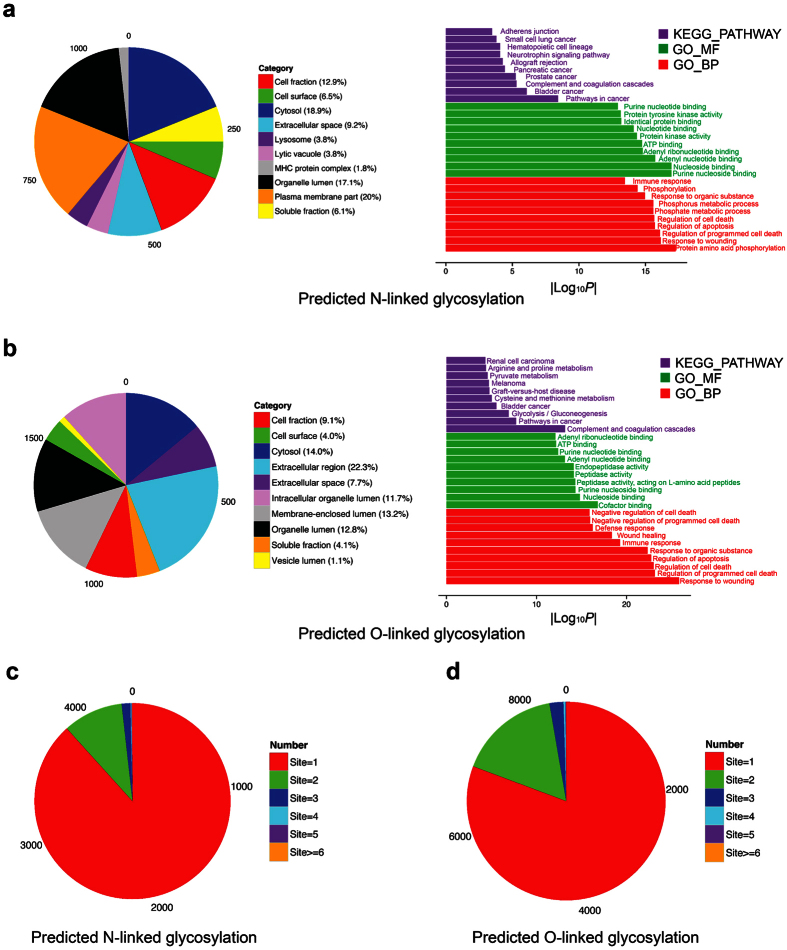
Functional enrichment analysis and classification of N-linked and O-linked glycoproteomes in terms of protein subcellular location, KEGG pathway, molecular function and biological process based on GO annotations. (**a**) Subcellular locations and GO terms enriched in N-linked glycosylated proteins. (**b**) Subcellular locations and GO terms enriched in O-linked glycosylated proteins. (**c,d**) Distributions of N-linked and O-linked glycosylated proteins categorized based on the numbers of predicted glycosylation sites.

To better understand the functional enrichment and systems impact of N- and O-linked glycosylation at the structural proteome level, we used the DAVID software^54^,^55^ to perform in-depth bioinformatics analysis of the significantly enriched gene ontology (GO), Kyoto Encyclopedia of Gene and Genomes (KEGG) pathways, and functional annotations in terms of cellular component (GO_CC), biological process (GO_BP), molecular function (GO_MF), and key functional pathways (KEGG_PATHWAY), for N- and O-linked glycosylated proteins, respectively. The overlap between the two lists of N- and O-linked glycosylated proteins indicates that some proteins were predicted to contain both N- and O-linked glycosylation sites. The top 10 significantly enriched GO_CC, GO_BP, GO_MF and KEGG_PATHWAY terms are displayed in Fig. 6a,b. We found that a suitably number of proteins were located within the “extracellular region” and “cytosol” (in terms of GO_CC). During their biogenesis extracellular proteins are first translocated into the endoplasmic reticulum where they may be subject to N-linked glycosylation^10^, and trafficked via the Golgi compartments where they may be subject to O-linked glycosylation^56^. The cytosol is another common “cellular component” for glycosylated proteins, being the sub-cellular compartment in which many O-GlcNAc transferases^57^ and glycosylated enzymes^1^ reside. We note that for most proteins there exist more than one subcellular location and/or cell component annotations. For example, the cellular component and subcellular location of a protein can be annotated as in the cytoplasm, membrane and nucleus. While this may sometimes reflect experimental difficulties in defining sub-cellular compartments, in at least some cases protein localization changes in response to cellular signals, either regulatory or in disease scenarios, such as is the case for mucin glycoproteins in human cancers, and other factors regulating cell death^58^. When performing statistical analysis of the GO term enrichment, such multi-location (i.e. “multi-component”) proteins will also be taken into account. It is of particular interest that both N- and O-linked glycosylated proteins were commonly enriched in several KEGG pathways involving complement and coagulation cascades, as well as bladder cancer. There also exist other cancer types that were specifically enriched for N-linked (e.g., pancreatic and prostate) and O-linked (melanoma and renal cell carcinoma) glycosylated proteins. Accordingly, several previous studies also outlined the roles of protein glycosylation and its implications in cancer pathways^6^, especially prostate cancer^59^, bladder cancer^60^ and pancreatic cancer^61^.

In terms of the biological processes, we found that regulation of cell death (*p* = 2.20 × 10^−16^ and *p* = 1.00 × 10^−23^ for N- and O-linked glycosylated proteins, respectively) and immune response (*p* = 3.50 × 10^−14^ and *p* = 5.30 × 10^−20^ for N- and O-linked glycosylated proteins, respectively) were two commonly enriched biological processes shared by N- and O-linked glycosylated proteins. This observation is consistent with a number of immunological studies suggesting that glycosylation plays an essential role in activating and maintaining the immune response^62^. Additionally, glycosylation was characterized as an important regulator for cell growth and death^63^, which has been confirmed by our GO-term enrichment analysis. Moreover, we found that phosphorylation (*p* = 5.50 × 10^−18^ for N-glycosylated proteins) and related processes were significantly enriched. This highlights the potential for large-scale cross-regulation between glycosylation and phosphorylation in related pathways as previously reported^64^.

Regarding molecular function, terms related to binding activity were commonly shared by N- and O-linked glycosylated proteins. For example, “purine nucleoside binding” (*p* = 1.10 × 10^−17^) and “cofactor binding” (*p* = 1.60 × 10^−17^) were the most significant GO_MF terms for N- and O-linked glycosylated proteins, respectively. Binding activities, such as nucleoside binding^65^, protein binding/inhibiting^1^,^66^, and adenosine triphosphate binding^67^,^68^ were experimentally validated as being closely associated with glycosylation. Additionally, glycosylation was implicated in the catalytic activities of dipeptidyl-peptidase IV^69^ and tripeptidyl-peptidase I^70^, both annotated as “peptidase activity” associated with O-linked glycosylated proteins in Fig. 6b. Analysing the distribution of glycosylated proteins classified based on the number of predicted N- and O-linked glycosylation sites (Fig. 6c,d) revealed that the majority of the glycoproteins contained only one predicted glycosylation site, while a limited number of proteins contained more than six glycosylation sites.

## Discussion

Glycosylation is a crucial and ubiquitous type of protein PTM by which carbohydrates are covalently attached to functional groups of a target protein. A better understanding of the most important determinants of protein glycosylation at both the sequence and structure levels required for highly accurate mapping of the human glycoproteome. In this study, we developed a novel bioinformatics tool termed *GlycoMine*^*struct*^ for improved prediction of N- and O-linked glycosylation. It utilizes a variety of complementary sequence-derived and structural features to enable accurate predictions of glycosylation. Using an efficient two-step feature-selection strategy, 14 and 11 optimal features at both the sequence and structural levels were systematically characterized as crucial features for N- and O-linked glycosylation prediction, respectively. The performance of *GlycoMine*^*struct*^ was extensively evaluated using both benchmark and independent test datasets. Five-fold cross-validation and independent testing showed that *GlycoMine*^*struct*^ outperformed NGlycPred, the only available N-linked glycosylation predictor incorporating structural information. Additionally, GO-term analysis revealed commonly and differentially enriched subcellular locations, biological processes, molecular functions, and functional pathways shared between the N- and O-linked glycoproteome. Furthermore, we applied *GlycoMine*^*struct*^ to accurately predict N- and O-linked glycosylation substrates and sites in the human structural proteome. Overall, this study provided a foundation for accurate prediction of the two important types of glycosylation sites in the human proteome. More generally, the techniques and framework of *GlycoMine*^*struct*^ should be also applicable to other types of PTM sites in proteins with available structural information.

A remaining limitation of the current *GlycoMine*^*struct*^ algorithm is that it cannot consider the stoichiometry of modification, i.e. the extent to which any given Asn, Ser or Thr residue will be modified with a glycan. There have been several studies for investigating the stoichiometries of protein phosphorylation^71^,^72^,^73^ and glycosylation^24^,^74^,^75^. However, to the best of our knowledge, there are no systematic datasets for quantitatively modified sites of both N- and O-linked glycosylation in association with the protein stoichiometry, which makes it very challenging to consider such knowledge into the glycosylation predictors at this stage. One note of hope in this regard comes from the case study of α-L-iduronidase where the consensus Asn-190 residue, which is known to be subject to only partial glycosylation^50^, was predicted as glycosylated by *GlycoMine*^*struct*^ but with a score below the prediction. Perhaps with sufficient data on sites subject to partial occupancy by glycan a dual threshold might be set on predictions to recover “high-stoichiometry” and “partial” extents of glycosylation in the predictor.

Another limitation is that the current algorithm does not consider the biosynthesis pathways as a feature during the model training process, due to the limited availably of annotated entries and the difficulty of extracting such annotations from other third-party databases. We anticipate that with the increasing availability of such biosynthesis pathway data particularly for O-linked glycosylation, further improvement of the performance of our algorithm will become possible.

As an implementation of *GlycoMine*^*struct*^, an online web server was developed to facilitate high-throughput prediction of N- and O-linked glycosylation sites in human proteins having available structural information. The server is configured using Tomcat 7 (Apache Software Foundation, Forest Hill, MD, USA) and JavaServer Pages (Sun Microsystems, Santa Clara, CA, USA) and is operated under the Linux environment with a 4-TB hard disk and 8 GB memory. The glycosylation site-prediction models used by the server were trained with OFSs on the complete training data used in this study. The server requires users to upload a protein structure file (a. pdb file is preferred), specify the chain name and glycosylation type and provide email addresses. Each submitted job normally takes 4 minutes to complete, and the server will send an email to users once the task is finished (see Supplementary Fig. S4 for the user interface and example prediction output). We hope that this novel approach along with the predicted N- and O-linked glycosylation sites from the human structural proteome address the concerns of the research community^29^ and provide a solid foundation for development of more accurate glycosylation-site predictors and prioritization of glycosylated candidates for follow-up functional validation.

## Methods

### Dataset construction

The annotations of C-, N-, and O-linked glycosylation sites were extracted from four major public resources, including UniProt^76^, PhosphoSitePlus^77^, SysPTM^78^, and O-GlycBase (version 6.0). Only experimentally verified glycosylation sites in the human proteins were retained^30^. To ensure the quality of the curated datasets, any glycosylation sites annotated as “Probable”, “Potential” or “By similarity” were discarded when extracting sequences from UniProt^30^. All remaining sequences were mapped to the PDB database^53^ using PSI-BLAST^79^. PDB entries were selected using the following criteria: (1) X-ray structures only, while nuclear magnetic resonance and electron microscopy structures were excluded; (2) X-ray resolution better than 2.5 Å; (3) structures with missing atoms were removed; and (4) the structure with the highest resolution was selected for protein sequences with more than one mapped PDB structure. The CD-HIT program^80^ was applied to cluster homologous sequences and reduce sequence redundancy at sequence-identity threshold of 70%^35^. We obtained 208 N-linked and 29 O-linked glycosylated PDB structures, which corresponded to 570 N-linked and 47 O-linked glycosylation sites, respectively. Initially, we also sought C-linked glycosylation sites within our frame of reference; however, as there was only one PDB structure containing C-linked glycosylation in the datasets, we removed this protein from our analysis and focused on N-linked and O-linked glycosylation prediction.

Regarding the selection of negative data, we extracted information on the relevant amino acid residues (i.e. Asn, Ser and Thr) that were not annotated as glycosylation sites, but that were present in those proteins that contain experimentally verified glycosylation sites. This effort to enhance the reliability of selection of negative sites is based on three criteria: (i) these proteins are biosynthetically relevant to the glycosylation machinery, in that they must be co-located with the relevant machinery i.e. in order to have one or more O-linked glycosylation sites the protein must share sub-cellular location together with an O-glycosidase, (ii) the expression of these glycoproteins must be temporally and developmentally coordinated with the expression of an appropriate glycosidase, and (iii) the experimental data validating glycosylation on at least one site of the given protein is the closest thing available to an experimental validation of non-glycosylation on the other potential sites. However, we also noticed that it was challenging to definitely determine whether the non-glycosylation sites would be glycosylated after being secreted. To the best of our knowledge, there is currently lack of sizable experimental datasets with such annotations.

Another important issue was highly imbalanced datasets, i.e., non-glycosylation sites greatly outnumbered glycosylation sites. If this imbalanced set had been used for model training, the trained models would be highly biased and classify each site in a protein as a non-glycosylation site. To address this imbalance, we used an under-sampling strategy, that enable all experimentally verified N- an O-linked glycosylation sites to be used as positive samples, while the same portion of amino acid residues (i.e., N, S and T) that had not been experimentally verified as glycosylation sites were randomly selected as negative samples from the positive PDB chains (this resulted in positive-to-negative ratio of 1:1). The datasets were further divided into two subsets consisting of benchmark and independent test datasets, which were ~20% of the size of the complete dataset. The benchmark dataset was used for performing five-fold cross-validation and feature selection, while the independent test dataset was used for validation of model performance.

### Feature extraction

A variety of sequence and structural features were calculated and extracted in this study. A full list of features can be found in the Supplementary Table S3.

#### Sequence features

AAindex: hydrophobicity, flexibility, polarity, and β-turn values were extracted from the AAindex database^81^.

Physicochemical properties: physicochemical properties of proteins were calculated using BioJava^82^; these properties included pK1 (-COOH), pK2 (-NH3 + ), pKR (R group), pI, hydropathy index, percentage occurrence in proteins, percentage of buried residues, average volume, accessible surface area, van der Waals volume, ranking of amino acid polarities, side-chain polarity, conformational preferences of amino acids (α-helix), and conformational preferences of amino acids (β-strand).

Position-specific scoring matrices (PSSMs): these were calculated by PSI-BLAST^79^ searches against UniRef90, with three iterations and e-value of 0.001^83^.

Residue-conservation score: conservation score was derived from the PSSM generated by PSI-BLAST^79^ and is defined as follows:


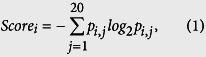


where *p*_*i,j*_ is the frequency of amino acid *j* at position *i*^30^.

#### Structural features

Surface accessibility: surface-accessible area of each protein was calculated by NACCESS^84^ using a probe of radius *=* 3 Å^35^. Five classes of surface-accessible area were contained in the output of NACCESS^84^ and used as structural features in this study, including all-atoms, non-polar side chains, polar side chains, total side chains and main chains.

Secondary structure: secondary structure features were calculated by DSSP^85^. These included ACC, phi, and psi, and were selected from the output of DSSP as the input features. ACC denotes the solvent accessibility of amino acid residue in terms of the number of water molecules in contact with the corresponding residue, while phi and psi represent two types of International Union of Pure and Applied Chemistry backbone-torsion angles.

Log-odds ratio: log-odds ratio^86^ is a statistical feature calculated by DiscoTope^87^.

Depth index: the PSAIA program^88^ was used to calculate a series of features for depth index, including the average depth index (denoted as ave_dpx), standard deviation of the depth index (sd_dpx), side-chain average depth index (s-ch_ave_dpx), and standard deviation of the side-chain depth index (sd_s-ch_dpx).

B-factor: for each residue, we extracted the B-factor scores of all atoms from protein structure files and calculated their average value^89^.

#### Feature window

The location of glycosylation sites may be influenced by surrounding residues at both the sequence and structure level. Therefore, we used sequence and structure windows to encode such features and capture potentially useful information.

Sequence window: to extract the sequence context information surrounding the glycosylation sites, we employed a local sliding window with 2*N* + 1 *=* 15 (*N* *=* 7, where *N* denotes the half-window size) residues to represent glycosylation sites. This was used in our previous work and proved to be effective^30^. In terms of feature nomenclature, each residue was named as P*X*, where *X* presents the *X-*th position of the feature in the local sliding window. The centered glycosylated residue was then denoted as P8. Accordingly, PSSM features, which have a total dimensionality of 15 × 20 *=* 300, were denoted as P1, P2, …, P300, respectively. Consequently, a total of 385 sequence-based features for each glycosylation and non-glycosylation site were obtained.

Structure window: we adopted a structure window to extract features of spatially neighbouring residues of a potential glycosylation site from protein structures^89^ using a sphere radius *R (R* *=* 10 Å). All spatially proximal residues were included in the structure window if the distance between any atoms of such residues and any atoms of the target residue of interest were less than a threshold, *R*. After extracting all 14 structural features from each of the spatial residues in the structure window, we then calculated the average values of all structural features for all residues involved within the structure window for each glycosylation/non-glycosylation residue. As a result, a total of 14 structural features for each glycosylation site were obtained.

### Feature selection

The proposed feature-encoding schemes led to a high-dimensionality feature vectors, requiring considerable computational time and memory to process. Meanwhile, the initial feature set may contain noisy, redundant and irrelevant features, which will have a potentially negative impact on model performance. In light of this, it was necessary to apply feature-selection methods to reduce the dimensionality of feature vectors by removing redundant and non-contributing features. We used a two-step feature-selection procedure to rank and select the most informative features.

#### Linear SVM-based feature selection

The first step of feature selection was performed using the linear SVM feature-selection method, which is competitive with traditional feature-selection methods, such as odds ratio and information gain^36^. For linear-kernel SVMs, the class predictor can be denoted as


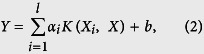


For a linear kernel,


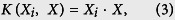


and the class predictor can be rewritten as


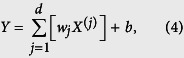


where


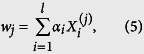


where the absolute value |*w*_*j*_| is used as the weight of a feature *j*. The larger the absolute value of a feature coefficient *w*_*j*_ is, the more useful the feature is for the classification^36^. We used LibSVM^90^ to calculate |*w*_*j*_|. The top ranked 300 features were then used as the optimal feature candidates (OFCs).

#### Incremental Feature Selection (IFS)

To determine the final optimal features from the OFCs at the second step of feature selection, an IFS strategy based on a random forest (RF)^91^ classifier was applied to the benchmark dataset by performing five-fold cross-validation to assess the relative importance and contribution of all OFCs. The IFS procedure can be briefly described as follows. First, it constructs *n (n* = |OFCs|) feature subsets by adding one feature at a time from OFCs to the candidate feature subset *F.* Then, the performance of the RF classifier that was trained based on the updated *F* in each round was evaluated using five-fold cross-validation to avoid over-fitting each time. This process was repeated for 20 rounds, and the average performance was calculated. The *i*-th feature subset is defined as *F* = {*f* | *f*_*1*_*, f*_*2*_*, …, f*_*i*_}^30^, where *f*_*i*_ is the *i*-th feature from the OFCs. As a result, the feature set with the highest area-under-the-curve (AUC) value amongst the 300 AUC values was selected as the optimal feature set (OFS).

#### Model training and performance evaluation

We employed the RF algorithm implemented in the R package^92^ to build glycosylation-site prediction models. RF is an ensemble machine-learning approach based on decision trees and has been successfully applied in many different tasks in protein bioinformatics, such as prediction of RNA-binding sites^93^, phosphorylation sites^94^, protease-cleavage sites^95^ and functional effects of single amino acid variants^96^. To evaluate the performance of RF classifiers, six performance measures were used, including sensitivity, specificity, precision, accuracy (ACC), the Matthews correlation coefficient (MCC), and AUC. Additionally, the receiver operating characteristic (ROC) curves were also generated, which plotted true-positive rate (TPR) against the false-positive rate (FPR). The ROC curves were drawn and the corresponding AUC values were calculated using the ROCR package^97^. Refer to the Supplemental Methods for a detailed description of these measures.

## Additional Information

**How to cite this article**: Li, F. *et al. GlycoMine*^*struct*^: a new bioinformatics tool for highly accurate mapping of the human N-linked and O-linked glycoproteomes by incorporating structural features. *Sci. Rep.* **6**, 34595; doi: 10.1038/srep34595 (2016).

## Supplementary Material

Supplementary Information

## Figures and Tables

**Table 1:** The selected optimal features for N-linked glycosylation.

Num.	Feature	Position	Software
**V1**	Normalized average hydrophobicity scales	P10	AAindex^81^
***V2***	*Absolute accessibility of non-polar side-chain*	*P1*	*NACCESS*^84^
**V3**	PSSM	P250	PSI-BLAST^79^
**V4**	PSSM	P235	PSI-BLAST^79^
***V5***	*Standard deviation of side-chain depth index*	*P1*	*PSAIA*^88^
**V6**	Conformational parameter of beta-turn	P10	AAindex^81^
**V7**	PSSM	P173	PSI-BLAST^79^
***V8***	*Absolute accessibility of main chain*	*P1*	*NACCESS*^84^
**V9**	Mean polarity	P8	AAindex^81^
***V10***	*Log-odds ratio*	*P1*	*DiscoTope*^87^
**V11**	Average flexibility indices	P7	AAindex^81^
**V12**	Mean polarity	P10	AAindex^81^
***V13***	*Absolute accessibility of all-atoms*	*P1*	*NACCESS*^84^
**V14**	PSSM	P274	PSI-BLAST^79^

Features highlighted in italic indicate structural features, while other features not highlighted are sequence-derived features or amino acid properties.

**Table 2:** The selected optimal features for O-linked glycosylation.

Num.	Feature	Position	Software
**V1**	Conformational parameter of beta-turn	P8	AAindex^81^
**V2**	PSSM	P38	PSI-BLAST^79^
***V3***	*B factor*	*P1*	*PDB file*^53^
**V4**	Normalized average hydrophobicity scales	P8	AAindex^81^
***V5***	*Standard deviation of side-chain depth index*	*P1*	*PSAIA*^88^
**V6**	PSSM	P293	PSI-BLAST^79^
**V7**	PSSM	P248	PSI-BLAST^79^
**V8**	PSSM	P8	PSI-BLAST^79^
**V9**	PSSM	P128	PSI-BLAST^79^
***V10***	*Absolute accessibility of main chain*	*P1*	*NACCESS*^84^
**V11**	Mean polarity	P8	AAindex^81^

Features highlighted in italic indicate structural features, while other features not highlighted are sequence-derived features or amino acid properties.
