# Supplementary material for: GlycoMinestruct: a new bioinformatics tool for highly accurate mapping of the human N-linked and O-linked glycoproteomes by incorporating structural features
Source: Sci Rep. 2016 Oct 6;6:34595. doi: 10.1038/srep34595 (PMC5052564; doi:10.1038/srep34595)
Supplement: Supplementary Information [file srep34595-s1.pdf]

Supplementary materials

GlycoMine^{struct}: a new bioinformatics tool for highly accurate mapping of the human N-linked and O-linked glycoproteomes by incorporating structural features

Fuyi Li[1,2,†], Chen Li[2,†], Jerico Revote[3], Yang Zhang[1,*], Geoffrey I. Webb[4], Jian Li[6], Jiangning Song[2,4,5,*] and Trevor Lithgow[6,*]

[1]College of Information Engineering, Northwest A&F University, Yangling 712100, China, [2]Infection and Immunity Program, Biomedicine Discovery Institute and Department of Biochemistry and Molecular Biology, Monash University, Melbourne, VIC 3800, Australia, [3]Monash Bioinformatics Platform, Monash University, Melbourne, VIC 3800, [4]Monash Centre for Data Science, Faculty of Information Technology, Monash University, Melbourne, VIC 3800, Australia, [5]National Engineering Laboratory for Industrial Enzymes and Key Laboratory of Systems Microbial Biotechnology, Tianjin Institute of Industrial Biotechnology, Chinese Academy of Sciences, Tianjin 300308, China and [6]Infection and Immunity Program, Biomedicine Discovery Institute and Department of Microbiology, Monash University, Melbourne, VIC 3800, Australia

[*]To whom correspondence should be addressed: zhangyang@nwsuaf.edu.cn; Jiangning.Song@monash.edu and Trevor.Lithgow@monash.edu.

[†]The two authors contributed equally to this work.

Supplemental methods

In this study, we used six measures, including Sensitivity (or true positive rate (TPR)), Specificity, Precision (PRE), Accuracy (ACC), Mathew's Correlation Coefficient (MCC), and the area under the curve (AUC) to evaluate the prediction performance of *GlycoMine^{struct}*. The first five measures are defined as follows:

Sensitivity:

$$\text{Sensitivity} = \text{TP}/(\text{TP} + \text{FN}),$$

Specificity:

$$\text{Specificity} = \text{TN}/(\text{TN} + \text{FP}),$$

Precision:

$$\text{Precision} = \text{TP}/(\text{TP} + \text{FP}),$$

Accuracy:

$$\text{Accuracy} = \frac{\text{TP} + \text{TN}}{\text{TP} + \text{TN} + \text{FP} + \text{FN}}$$

Mathew's Correlation Coefficient (MCC):

$$\mathbf{MCC} = \frac{\mathbf{TP}\times\mathbf{TN} - \mathbf{FP}\times\mathbf{FN}}{\sqrt{(\mathbf{TP} + \mathbf{FP})(\mathbf{TP} + \mathbf{FN})(\mathbf{TN} + \mathbf{FP})(\mathbf{TN} + \mathbf{FN})}}$$

where *TP*, *TN*, *FP* and *FN* represents the numbers of true positives, true negatives, false positives and false negatives, respectively.

Supplemental results

Analysis of significantly enriched Gene Ontology (GO) terms for N- and O-glycosylated proteins

We collected GO terms for all the N- and O-glycosylated proteins in our benchmark dataset and conducted analyses of significantly enriched GO terms for both types of glycosylated proteins. The GO term annotations were extracted from the UniProt database[1] using the identifier for each protein. For statistical analysis, for each type of glycosylation, we prepared two datasets, i.e. the positive dataset (with glycosylated proteins) and the background set (from human proteins). We performed the two-side hypergeometric test to find significantly enriched GO terms from the positive set compared with the background set. The results for N- and O-glycosylation are respectively shown in **Supplementary Tables S4 and S5**. The GO term annotations involve three subcategories, namely cellular component (CC), molecular function (MF) and biological process (BP). For CC, it is clear from **Supplementary Tables S4 and S5** that O-glycosylated proteins possess more GO terms (110 GO terms) compared with N-glycosylated proteins (17 GO terms). 17 significantly enrich GO terms for N-glycosylated proteins include "Endoplasmic reticulum", "Cytosol", "Mitochondrion", "Mitochondrial matrix", "Centrosome", "Spindle pole", "Golgi apparatus", "Membrane", "Cell surface", "Extracellular region", "Integral component of membrane", "Lamellipodium", "Extracellular space", "Endoplasmic reticulum membrane", "Endolysosome membrane", "Beta-catenin destruction complex", and "Endolysosome lumen". While for O-linked glycosylation, the top ten significantly enriched GO terms are "ATP binding", "Proteolysis", "Negative regulation of apoptotic process", "Glycolytic process", and "Cellular response to lipopolysaccharide".

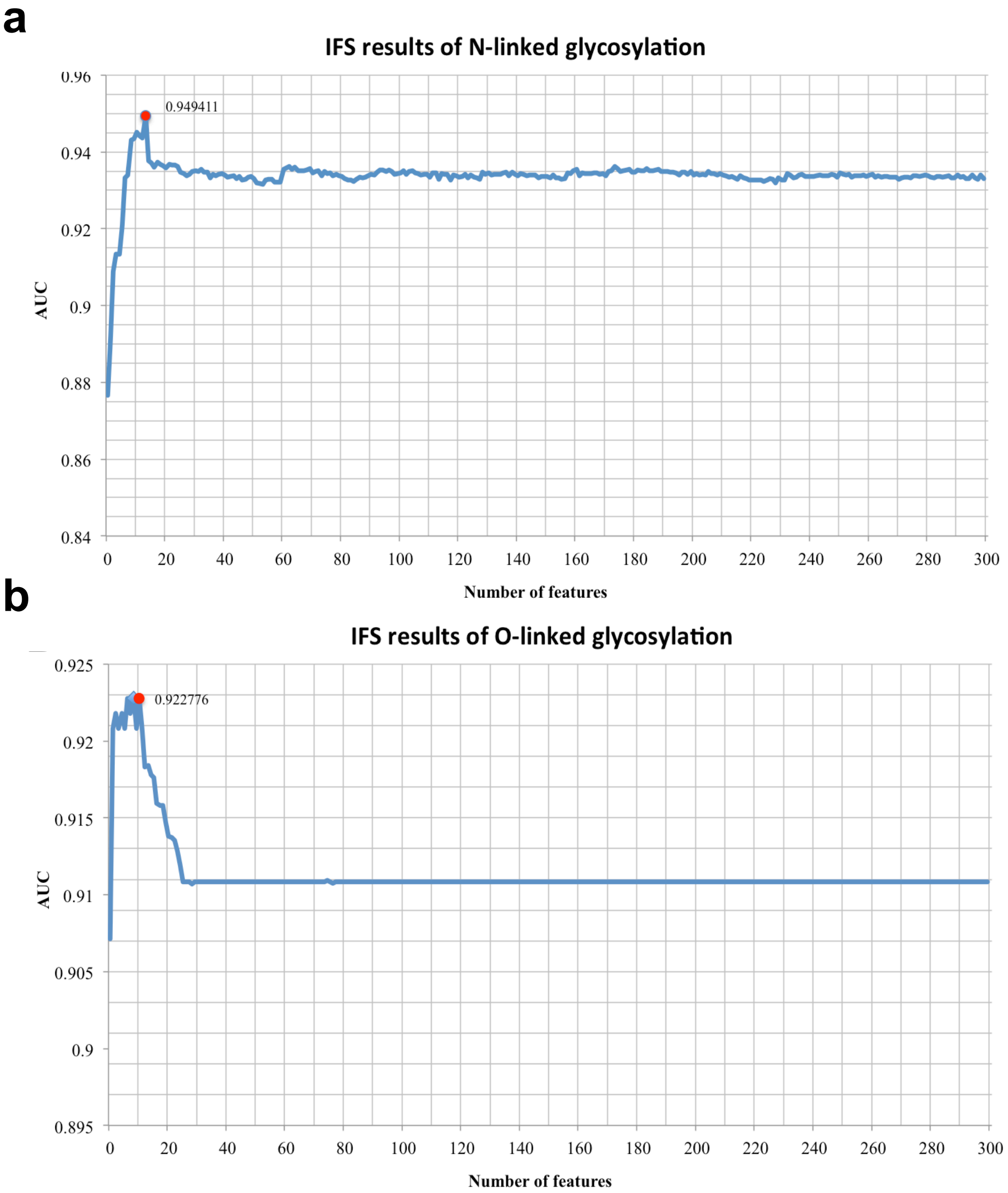

Supplementary Figure S1. IFS curves for the prediction of (**a**) N- and (**b**) O-linked glycosylation sites. The values highlighted in the graph correspond to the highest AUC scores obtained by IFS for N- and O-linked glycosylation, respectively.

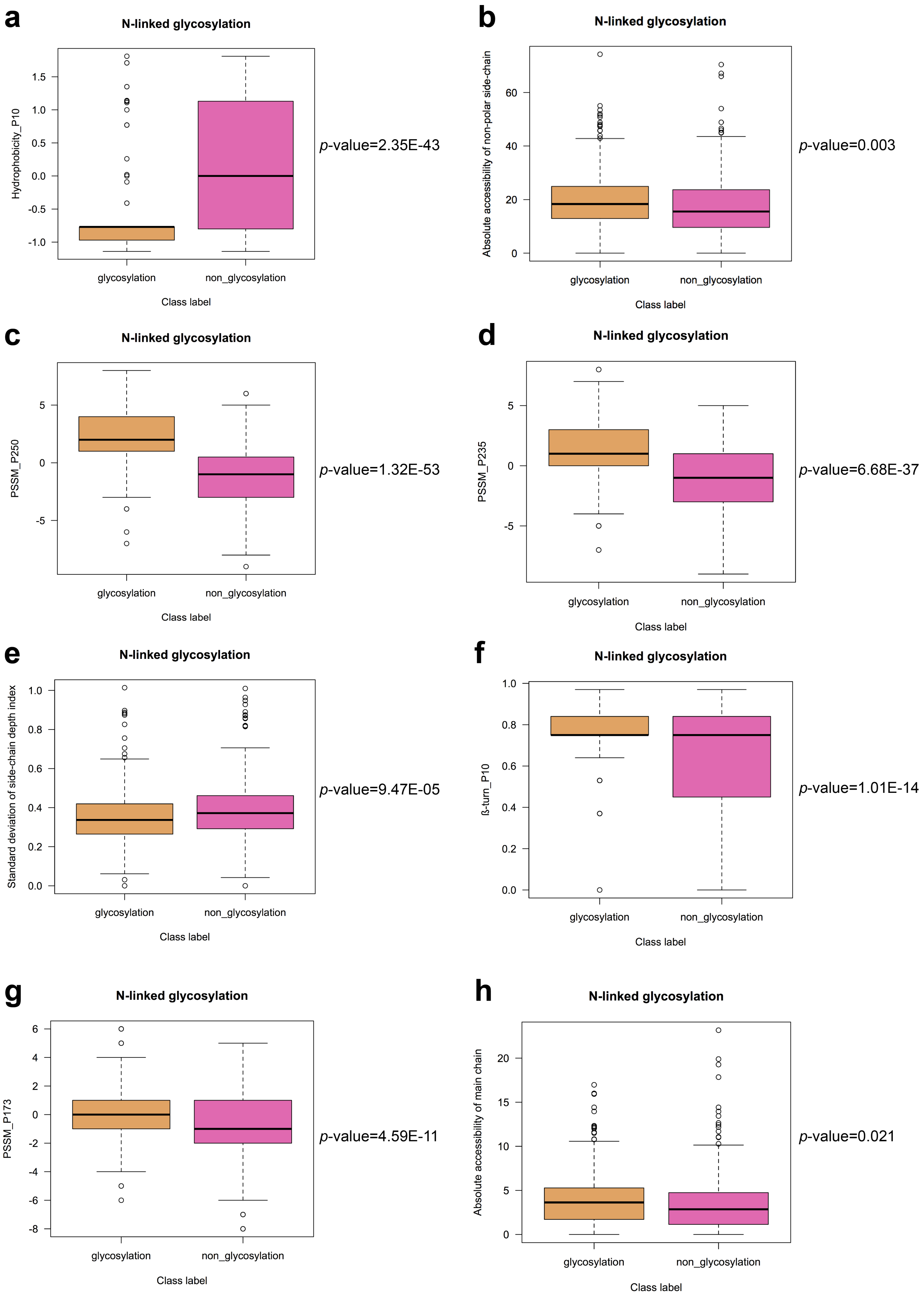
a
N-linked glycosylation
Hydrophobicity_P10
glycosylation
non_glycosylation
Class label
p-value=2.35E-43
b
N-linked glycosylation
Absolute accessibility of non-polar side-chain
glycosylation
non_glycosylation
Class label
p-value=0.003
c
N-linked glycosylation
PSSM_P250
glycosylation
non_glycosylation
Class label
p-value=1.32E-53
d
N-linked glycosylation
PSSM_P235
glycosylation
non_glycosylation
Class label
p-value=6.68E-37
e
N-linked glycosylation
Standard deviation of side-chain depth index
glycosylation
non_glycosylation
Class label
p-value=9.47E-05
f
N-linked glycosylation
ß-turn_P10
glycosylation
non_glycosylation
Class label
p-value=1.01E-14
g
N-linked glycosylation
PSSM_P173
glycosylation
non_glycosylation
Class label
p-value=4.59E-11
h
N-linked glycosylation
Absolute accessibility of main chain
glycosylation
non_glycosylation
Class label
p-value=0.021

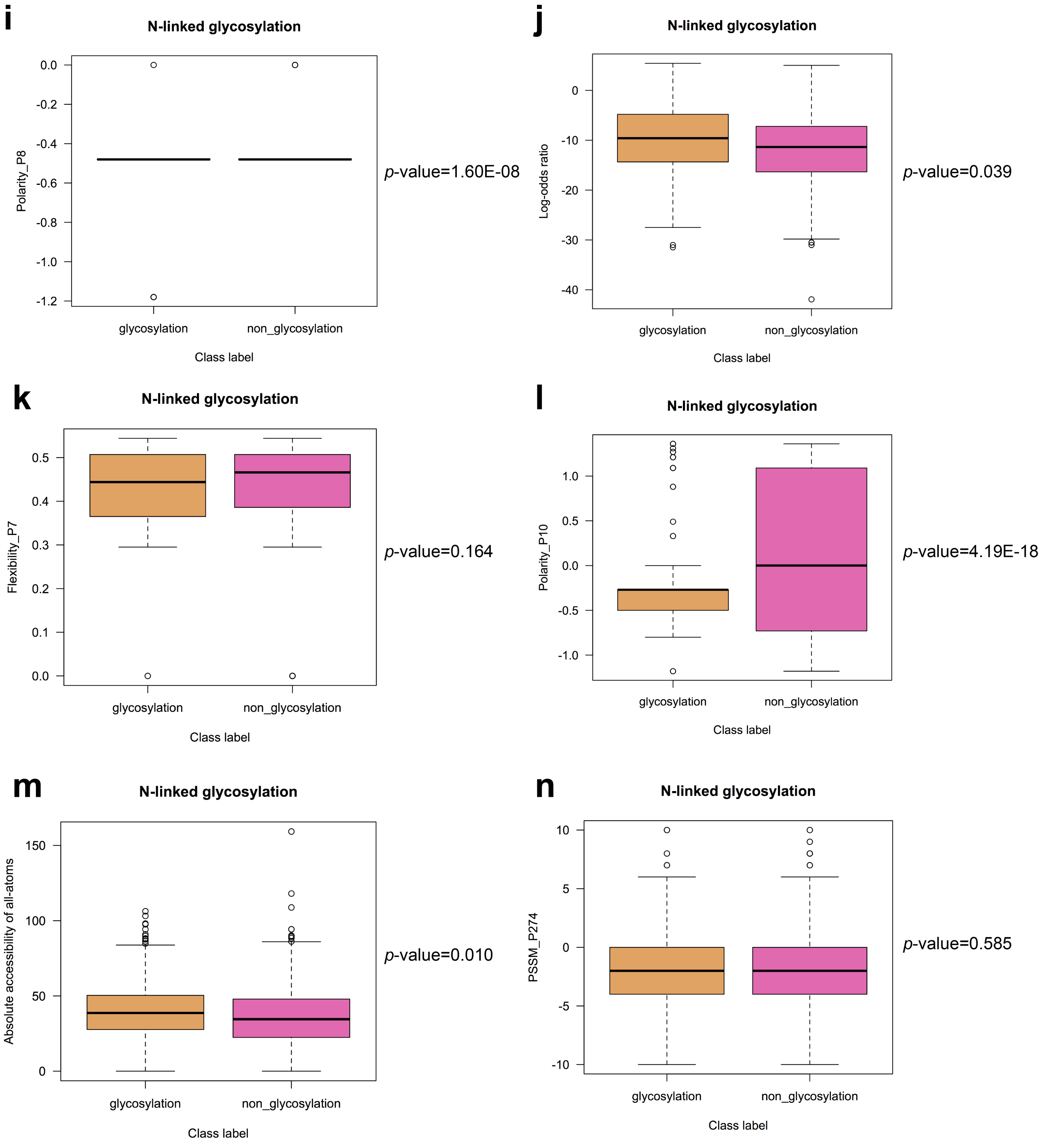

Supplementary Figure S2. Boxplots showing the significant difference in feature value distributions of the optimal sequence and structural features listed in Table 1 for N-linked glycosylation prediction.

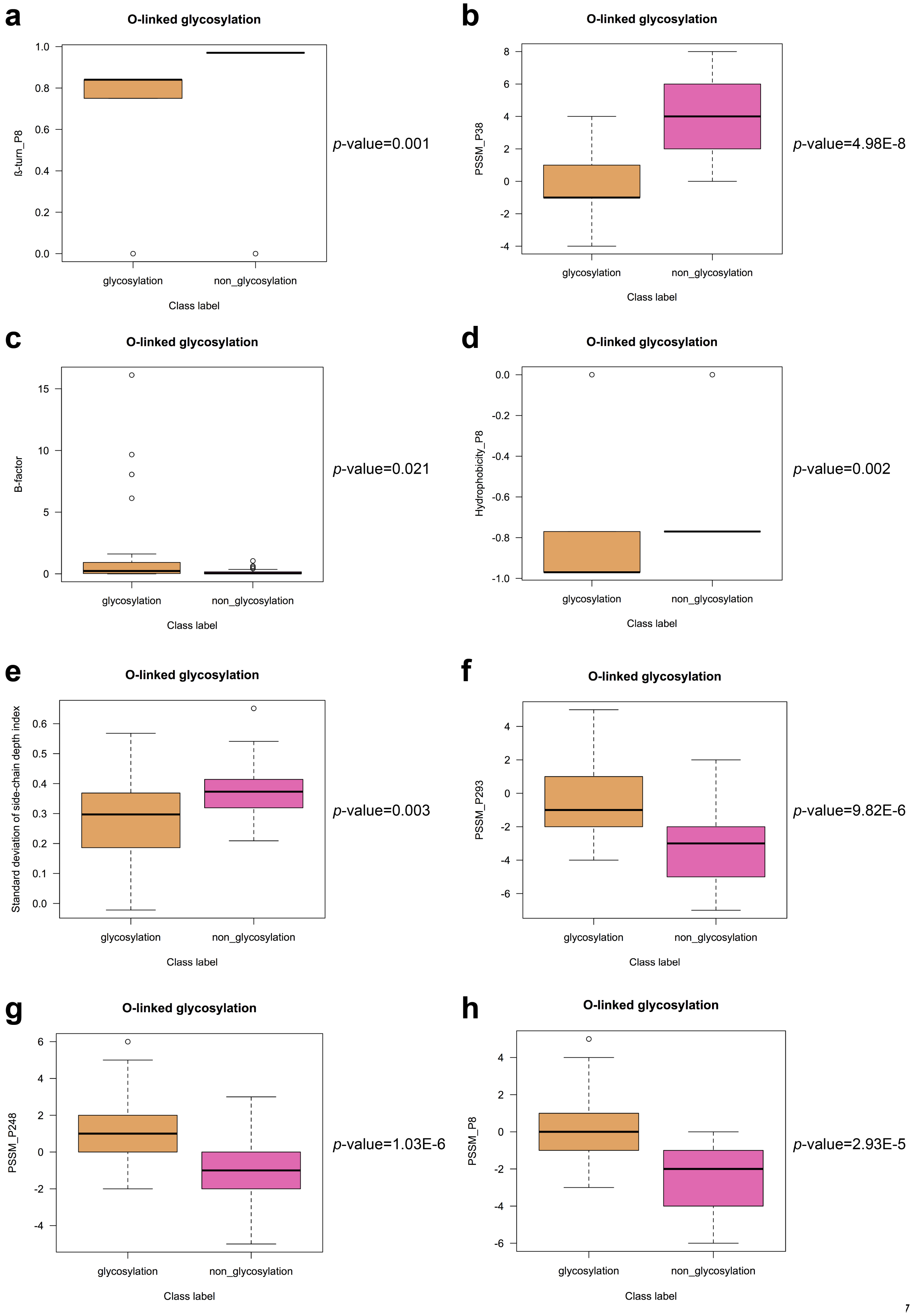

a
O-linked glycosylation
ß-turn_P8
1.0
0.8
0.6
0.4
0.2
0.0
glycosylation
non_glycosylation
Class label
p-value=0.001
b
O-linked glycosylation
PSSM_P38
8
6
4
2
0
-2
-4
glycosylation
non_glycosylation
Class label
p-value=4.98E-8
c
O-linked glycosylation
B-factor
15
10
5
0
glycosylation
non_glycosylation
Class label
p-value=0.021
d
O-linked glycosylation
Hydrophobicity_P8
0.0
-0.2
-0.4
-0.6
-0.8
-1.0
glycosylation
non_glycosylation
Class label
p-value=0.002
e
O-linked glycosylation
Standard deviation of side-chain depth index
0.6
0.5
0.4
0.3
0.2
0.1
0.0
glycosylation
non_glycosylation
Class label
p-value=0.003
f
O-linked glycosylation
PSSM_P293
4
2
0
-2
-4
-6
glycosylation
non_glycosylation
Class label
p-value=9.82E-6
g
O-linked glycosylation
PSSM_P248
6
4
2
0
-2
-4
glycosylation
non_glycosylation
Class label
p-value=1.03E-6
h
O-linked glycosylation
PSSM_P8
4
2
0
-2
-4
-6
glycosylation
non_glycosylation
Class label
p-value=2.93E-5

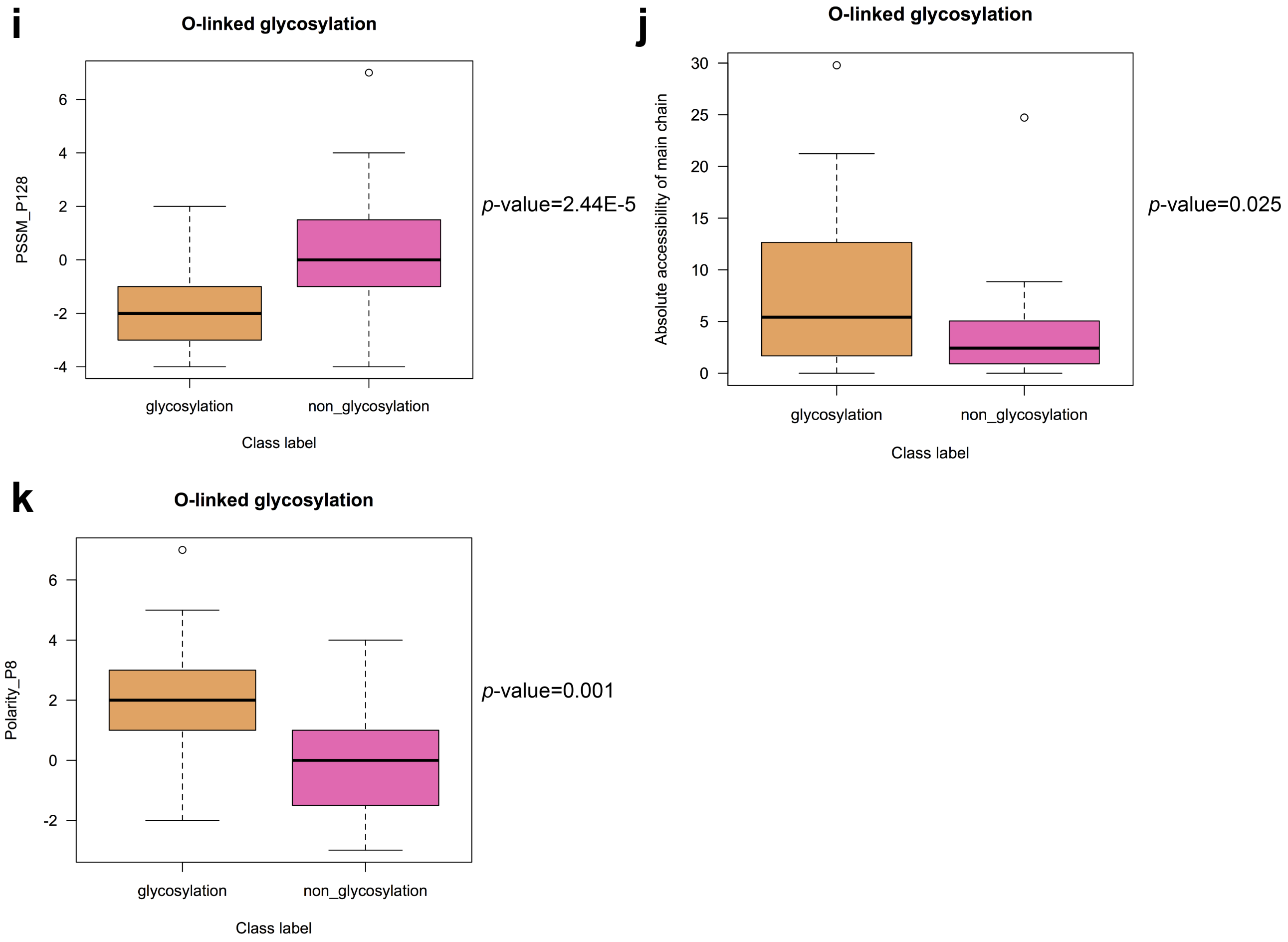

Supplementary Figure S3. Boxplots showing significant difference in feature value distributions of the optimal sequence and structural features listed in Table 2 for O-linked glycosylation prediction.

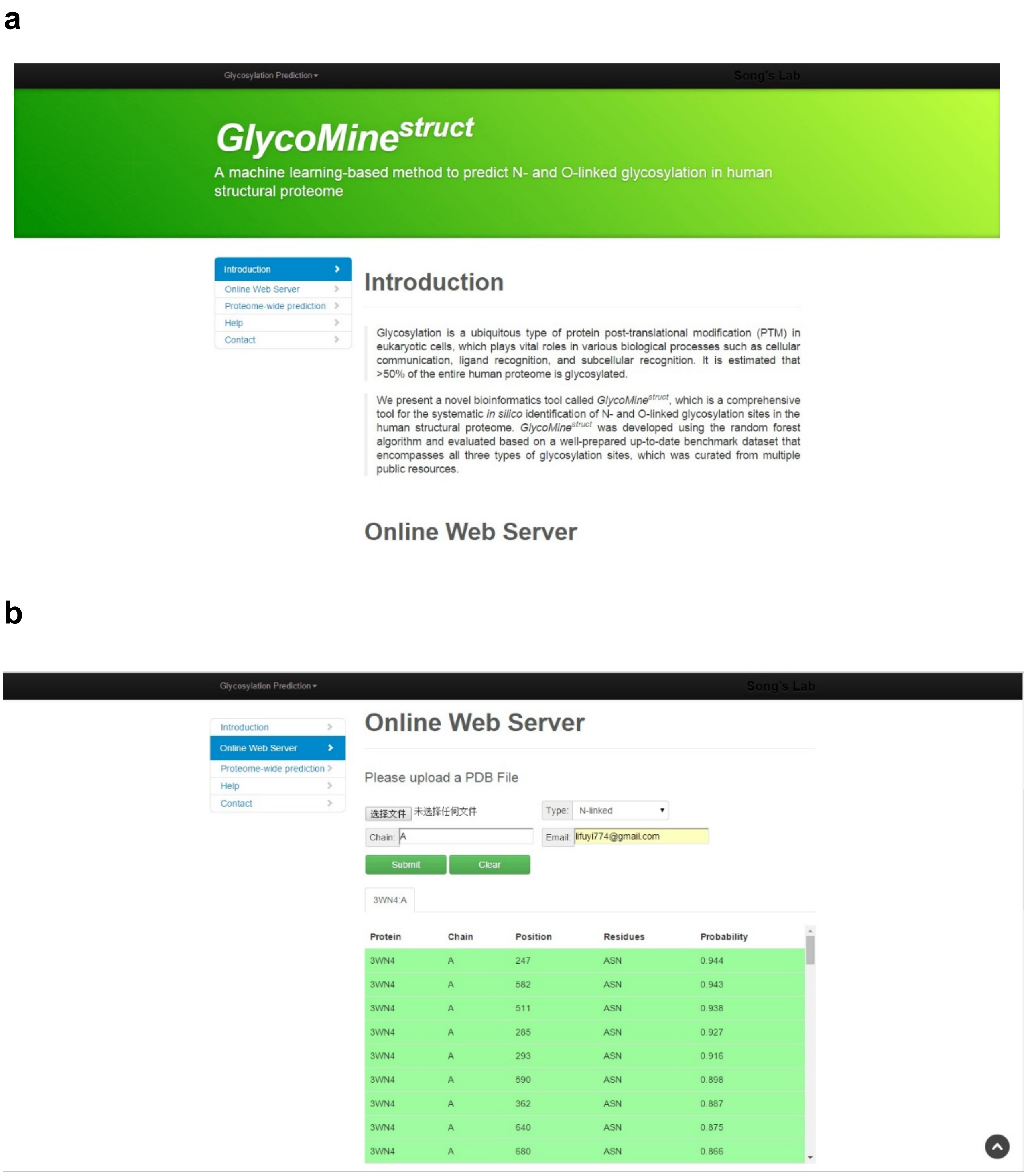

Supplementary Figure S4. Screenshots of *GlycoMine^{struct}* web server. (**a**) The user interface and (**b**) Display of the prediction output.

Supplementary Table S1. The prediction performance of N- and O-linked glycosylation sites based on 5-fold cross-validation and independent tests. The performance was evaluated using six measures including AUC, MCC, ACC, Sensitivity, Specificity, and Precision.

			**AUC**	**MCC**	**ACC**	**Sensitivity**	**Specificity**	**Precision**
**All OFS features**	**Benchmark**	N-linked	0.949	0.838	0.919	0.936	0.901	0.912
		O-linked	0.923	0.814	0.906	0.871	0.939	0.931
	**Independent**	N-linked	0.941	0.830	0.912	0.972	0.853	0.867
		O-linked	0.922	0.805	0.895	0.778	0.979	0.972
**Structural features only**	**Benchmark**	N-linked	0.911	0.734	0.865	0.823	0.911	0.910
		O-linked	0.836	0.660	0.828	0.871	0.788	0.794
**Sequence features only**	**Benchmark**	N-linked	0.880	0.695	0.846	0.811	0.883	0.884
		O-linked	0.814	0.547	0.766	0.871	0.667	0.711
**NGlycPred**	**Independent**	N-linked	0.804	0.685	0.840	0.785	0.896	0.884

Supplementary Table S2. Statistical summary of the proteome-wide prediction of N- and O-linked glycosylation sites at the 99% specificity level. The prediction was performed for the whole human structural proteome with a total of 20538 proteins.

**Glycosylation Type**	**Number of predicted glycosylated proteins**	**Number of predicted glycosylation sites**
N-linked	3386	4996
O-linked	5298	10529

Supplementary Table S3. Lists of all initial features explored in this study. (**a**) Sequence-derived features and (**b**) Structural features.

(**a**)

**Feature type**	**Description**	**Software**	**Number of features**
AAindex	The hydrophobicity score, flexibility score, polarity score and ß-turn score	AAindex[2]	4×15=60
Physicochemical properties	14 physicochemical properties	BioJava[3]	14
PSSMs	Position-specific scoring matrices	PSI-BLAST[4]	20×15=300
Residue conversation score	Residue conversation	PSI-BLAST[4]	1×15=15

(**b**)

**Feature type**	**Description**	**Software**	**Number of features**
Surface accessibility	All-atoms, total-side, main-chain, non-polar and all-polar accessibilities	NACCESS[5]	5
Secondary structure	ACC: the number of water molecules in contact with corresponding residue;<br>PHI and PSI: two IUPAC peptide backbone torsion angles.	DSSP[6]	3
Log-odds ratio	Log-odds ratio is a statistical feature	DiscoTope[7]	1
Depth Index	The average depth, standard deviation of depth index, side-chain average depth index and standard deviation of side-chain depth index	PSAIA[8]	4
B-factor	The average B-factor of all the atoms in this residue	PDB file[9]	1

Supplementary Table S4. Significantly enriched GO terms for N-linked glycosylated proteins.

Type	ID	Term in the positive	Term in the human proteome	P-value	Term
CC	GO:0005783	17	120	1.72E-3	Endoplasmic reticulum
	GO:0005829	97	1106	7.07E-4	Cytosol
	GO:0005739	32	271	6.50E-4	Mitochondrion
	GO:0005759	13	95	6.86E-3	Mitochondrial matrix
	GO:0005813	12	86	7.82E-3	Centrosome
	GO:0000922	6	27	6.56E-3	Spindle pole
	GO:0005794	20	176	7.63E-3	Golgi apparatus
	GO:0016020	17	122	2.03E-3	Membrane
	GO:0009986	16	132	8.82E-3	Cell surface
	GO:0005576	36	322	8.24E-4	Extracellular region
	GO:0016021	31	306	6.27E-3	Integral component of membrane
	GO:0030027	8	47	9.02E-3	Lamellipodium
	GO:0005615	33	327	5.35E-3	Extracellular space
	GO:0005789	14	109	8.58E-3	Endoplasmic reticulum membrane
	GO:0036020	2	2	4.62E-3	Endolysosome membrane
	GO:0030877	3	7	8.28E-3	Beta-catenin destruction complex
	GO:0036021	3	4	1.17E-3	Endolysosome lumen
MF	GO:0005524	65	537	5.79E-7	ATP binding
	GO:0004674	17	129	3.46E-3	Protein serine/threonine kinase activity
	GO:0009055	12	68	1.28E-3	Electron carrier activity
	GO:0004252	10	63	6.24E-3	Serine-type endopeptidase activity
	GO:0008201	9	55	7.53E-3	Heparin binding
	GO:0008144	6	28	7.79E-3	Drug binding
	GO:0005506	10	66	8.37E-3	Iron ion binding
	GO:0016887	4	13	8.06E-3	Atpase activity
	GO:0004222	7	34	5.28E-3	Metalloendopeptidase activity
	GO:0004716	4	8	1.12E-3	Receptor signaling protein tyrosine kinase

					activity
	GO:0004197	6	25	4.51E-3	Cysteine-type endopeptidase activity
	GO:0005149	2	2	4.62E-3	Interleukin-1 receptor binding
	GO:0005536	3	5	2.72E-3	Glucose binding
	GO:0043169	5	16	2.89E-3	Cation binding
	GO:0016812	2	2	4.62E-3	Hydrolase activity, acting on Carbon-nitrogen (but not peptide) bonds, in cyclic amides
	GO:0008013	3	6	5.07E-3	Beta-catenin binding
	GO:0004029	3	6	5.07E-3	Aldehyde dehydrogenase (NAD) activity
	GO:0016303	3	4	1.17E-3	1-phosphatidylinositol-3-kinase activity
	GO:0015038	2	2	4.62E-3	Glutathione disulfide oxidoreductase activity
	GO:0046934	2	2	4.62E-3	Phosphatidylinositol-4,5-bisphosphate 3-kinase activity
BP	GO:0045087	26	200	5.07E-4	Innate immune response
	GO:0043066	32	212	7.23E-6	Negative regulation of apoptotic process
	GO:0006508	28	170	5.21E-6	Proteolysis
	GO:0006006	7	37	8.21E-3	Glucose metabolic process
	GO:0070207	4	8	1.12E-3	Protein homotrimerization
	GO:0044281	26	198	4.38E-4	Small molecule metabolic process
	GO:0007409	6	18	7.69E-4	Axonogenesis
	GO:0046777	13	94	6.34E-3	Protein autophosphorylation
	GO:0002250	4	11	4.28E-3	Adaptive immune response
	GO:0001816	4	9	1.88E-3	Cytokine production
	GO:0071222	8	29	4.27E-4	Cellular response to lipopolysaccharide
	GO:0045080	3	7	8.28E-3	Positive regulation of chemokine biosynthetic process
	GO:0042493	16	133	9.36E-3	Response to drug
	GO:0030168	15	111	4.52E-3	Platelet activation
	GO:0002576	9	55	7.53E-3	Platelet degranulation
	GO:0006935	5	21	9.49E-3	Chemotaxis
	GO:0071260	7	33	4.50E-3	Cellular response to mechanical stimulus

GO:0045078	3	6	5.07E-3	Positive regulation of interferon-gamma biosynthetic process
GO:0006096	8	25	1.42E-4	Glycolytic process
GO:0005980	3	7	8.28E-3	Glycogen catabolic process
GO:0046686	5	14	1.52E-3	Response to cadmium ion
GO:0000281	3	7	8.28E-3	Mitotic cytokinesis
GO:0007160	7	26	1.12E-3	Cell-matrix adhesion
GO:0045780	3	7	8.28E-3	Positive regulation of bone resorption
GO:0050435	3	5	2.72E-3	Beta-amyloid metabolic process
GO:0007040	4	8	1.12E-3	Lysosome organization
GO:0007494	2	2	4.62E-3	Midgut development
GO:0060444	4	8	1.12E-3	Branching involved in mammary gland duct morphogenesis
GO:0030316	4	11	4.28E-3	Osteoclast differentiation
GO:0033628	3	7	8.28E-3	Regulation of cell adhesion mediated by integrin
GO:0002437	3	7	8.28E-3	Inflammatory response to antigenic stimulus
GO:0032611	2	2	4.62E-3	Interleukin-1 beta production
GO:0042346	4	11	4.28E-3	Positive regulation of NF-kappab import into nucleus
GO:0003073	2	2	4.62E-3	Regulation of systemic arterial blood pressure
GO:0030207	3	5	2.72E-3	Chondroitin sulfate catabolic process
GO:0045356	2	2	4.62E-3	Positive regulation of interferon-alpha biosynthetic process
GO:0060740	3	6	5.07E-3	Prostate gland epithelium morphogenesis
GO:0042733	4	9	1.88E-3	Embryonic digit morphogenesis
GO:0061198	2	2	4.62E-3	Fungiform papilla formation
GO:0060789	3	4	1.17E-3	Hair follicle placode formation
GO:0010042	2	2	4.62E-3	Response to manganese ion
GO:0071372	3	5	2.72E-3	Cellular response to follicle-stimulating hormone stimulus

GO:0003420	2	2	4.62E-3	Regulation of growth plate cartilage chondrocyte proliferation
GO:0070527	3	4	1.17E-3	Platelet aggregation
GO:0014909	3	4	1.17E-3	Smooth muscle cell migration
GO:0042117	2	2	4.62E-3	Monocyte activation
GO:0010641	2	2	4.62E-3	Positive regulation of platelet-derived growth factor receptor signaling pathway
GO:0050927	3	5	2.72E-3	Positive regulation of positive chemotaxis
GO:0035116	4	11	4.28E-3	Embryonic hindlimb morphogenesis
GO:0032331	3	6	5.07E-3	Negative regulation of chondrocyte differentiation
GO:0033146	2	2	4.62E-3	Regulation of intracellular estrogen receptor signaling pathway
GO:0050790	3	4	1.17E-3	Regulation of catalytic activity
GO:0051131	2	2	4.62E-3	Chaperone-mediated protein complex assembly
GO:0019953	2	2	4.62E-3	Sexual reproduction
GO:0055118	2	2	4.62E-3	Negative regulation of cardiac muscle contraction
GO:0048025	2	2	4.62E-3	Negative regulation of mrna splicing, via spliceosome
GO:0009966	3	4	1.17E-3	Regulation of signal transduction
GO:0061324	2	2	4.62E-3	Canonical Wnt signaling pathway involved in positive regulation of cardiac outflow tract cell proliferation
GO:0003344	2	2	4.62E-3	Pericardium morphogenesis

Supplementary Table S5. Significantly enriched GO terms for O-linked glycosylated proteins.

Type	ID	Term in the positive	Term in the human proteome	P-value	Term
CC	GO:0005783	17	120	1.72E-03	Endoplasmic reticulum
	GO:0005829	97	1106	7.07E-04	Cytosol
	GO:0005739	32	271	6.50E-04	Mitochondrion
	GO:0005759	13	95	6.86E-03	Mitochondrial matrix
	GO:0000922	6	27	6.56E-03	Spindle pole
	GO:0005794	20	176	7.63E-03	Golgi apparatus
	GO:0016020	17	122	2.03E-03	Membrane
	GO:0009986	16	132	8.82E-03	Cell surface
	GO:0005576	36	322	8.24E-04	Extracellular region
	GO:0016021	31	306	6.27E-03	Integral component of membrane
	GO:0030027	8	47	9.02E-03	Lamellipodium
	GO:0005615	33	327	5.35E-03	Extracellular space
	GO:0005789	14	109	8.58E-03	Endoplasmic reticulum membrane
	GO:0036020	2	2	4.62E-03	Endolysosome membrane
	GO:0030877	3	7	8.28E-03	Beta-catenin destruction complex
	GO:0036021	3	4	1.17E-03	Endolysosome lumen
	GO:0005524	65	537	5.79E-07	ATP binding
	GO:0004674	17	129	3.46E-03	Protein serine/threonine kinase activity
	GO:0009055	12	68	1.28E-03	Electron carrier activity
	GO:0004252	10	63	6.24E-03	Serine-type endopeptidase activity
	GO:0008201	9	55	7.53E-03	Heparin binding
	GO:0008144	6	28	7.79E-03	Drug binding
	GO:0005506	10	66	8.37E-03	Iron ion binding
	GO:0016887	4	13	8.06E-03	Atpase activity
	GO:0004222	7	34	5.28E-03	Metalloendopeptidase activity
	GO:0004716	4	8	1.12E-03	Receptor signaling protein tyrosine kinase activity

GO:0004197	6	25	4.51E-03	Cysteine-type endopeptidase activity
GO:0005149	2	2	4.62E-03	Interleukin-1 receptor binding
GO:0005536	3	5	2.72E-03	Glucose binding
GO:0043169	5	16	2.89E-03	Cation binding
GO:0016812	2	2	4.62E-03	Hydrolase activity, acting on Carbon-nitrogen (but not peptide) bonds, in cyclic amides
GO:0008013	3	6	5.07E-03	Beta-catenin binding
GO:0004029	3	6	5.07E-03	Aldehyde dehydrogenase (NAD) activity
GO:0016303	3	4	1.17E-03	1-phosphatidylinositol-3-kinase activity
GO:0015038	2	2	4.62E-03	Glutathione disulfide oxidoreductase activity
GO:0046934	2	2	4.62E-03	Phosphatidylinositol-4,5-bisphosphate 3-kinase activity
GO:0045087	26	200	5.07E-04	Innate immune response
GO:0043066	32	212	7.23E-06	Negative regulation of apoptotic process
GO:0006508	28	170	5.21E-06	Proteolysis
GO:0006006	7	37	8.21E-03	Glucose metabolic process
GO:0070207	4	8	1.12E-03	Protein homotrimerization
GO:0044281	26	198	4.38E-04	Small molecule metabolic process
GO:0007409	6	18	7.69E-04	Axonogenesis
GO:0046777	13	94	6.34E-03	Protein autophosphorylation
GO:0002250	4	11	4.28E-03	Adaptive immune response
GO:0001816	4	9	1.88E-03	Cytokine production
GO:0071222	8	29	4.27E-04	Cellular response to lipopolysaccharide
GO:0045080	3	7	8.28E-03	Positive regulation of chemokine biosynthetic process
GO:0042493	16	133	9.36E-03	Response to drug
GO:0030168	15	111	4.52E-03	Platelet activation
GO:0002576	9	55	7.53E-03	Platelet degranulation
GO:0006935	5	21	9.49E-03	Chemotaxis
GO:0071260	7	33	4.50E-03	Cellular response to mechanical stimulus

GO:0045078	3	6	5.07E-03	Positive regulation of interferon-gamma biosynthetic process
GO:0006096	8	25	1.42E-04	Glycolytic process
GO:0005980	3	7	8.28E-03	Glycogen catabolic process
GO:0046686	5	14	1.52E-03	Response to cadmium ion
GO:0000281	3	7	8.28E-03	Mitotic cytokinesis
GO:0007160	7	26	1.12E-03	Cell-matrix adhesion
GO:0045780	3	7	8.28E-03	Positive regulation of bone resorption
GO:0050435	3	5	2.72E-03	Beta-amyloid metabolic process
GO:0007040	4	8	1.12E-03	Lysosome organization
GO:0007494	2	2	4.62E-03	Midgut development
GO:0060444	4	8	1.12E-03	Branching involved in mammary gland duct morphogenesis
GO:0030316	4	11	4.28E-03	Osteoclast differentiation
GO:0033628	3	7	8.28E-03	Regulation of cell adhesion mediated by integrin
GO:0002437	3	7	8.28E-03	Inflammatory response to antigenic stimulus
GO:0032611	2	2	4.62E-03	Interleukin-1 beta production
GO:0042346	4	11	4.28E-03	Positive regulation of NF-kappab import into nucleus
GO:0003073	2	2	4.62E-03	Regulation of systemic arterial blood pressure
GO:0030207	3	5	2.72E-03	Chondroitin sulfate catabolic process
GO:0045356	2	2	4.62E-03	Positive regulation of interferon-alpha biosynthetic process
GO:0060740	3	6	5.07E-03	Prostate gland epithelium morphogenesis
GO:0042733	4	9	1.88E-03	Embryonic digit morphogenesis
GO:0061198	2	2	4.62E-03	Fungiform papilla formation
GO:0060789	3	4	1.17E-03	Hair follicle placode formation
GO:0010042	2	2	4.62E-03	Response to manganese ion
GO:0071372	3	5	2.72E-03	Cellular response to follicle-stimulating

				hormone stimulus
GO:0003420	2	2	4.62E-03	Regulation of growth plate cartilage chondrocyte proliferation
GO:0070527	3	4	1.17E-03	Platelet aggregation
GO:0014909	3	4	1.17E-03	Smooth muscle cell migration
GO:0042117	2	2	4.62E-03	Monocyte activation
GO:0010641	2	2	4.62E-03	Positive regulation of platelet-derived growth factor receptor signaling pathway
GO:0050927	3	5	2.72E-03	Positive regulation of positive chemotaxis
GO:0035116	4	11	4.28E-03	Embryonic hindlimb morphogenesis
GO:0032331	3	6	5.07E-03	Negative regulation of chondrocyte differentiation
GO:0050790	3	4	1.17E-03	Regulation of catalytic activity
GO:0051131	2	2	4.62E-03	Chaperone-mediated protein complex assembly
GO:0019953	2	2	4.62E-03	Sexual reproduction
GO:0055118	2	2	4.62E-03	Negative regulation of cardiac muscle contraction
GO:0048025	2	2	4.62E-03	Negative regulation of mrna splicing, via spliceosome
GO:0009966	3	4	1.17E-03	Regulation of signal transduction
GO:0061324	2	2	4.62E-03	Canonical Wnt signaling pathway involved in positive regulation of cardiac outflow tract cell proliferation
GO:0003344	2	2	4.62E-03	Pericardium morphogenesis
GO:0005783	17	120	1.72E-03	Endoplasmic reticulum
GO:0005829	97	1106	7.07E-04	Cytosol
GO:0005739	32	271	6.50E-04	Mitochondrion
GO:0005759	13	95	6.86E-03	Mitochondrial matrix
GO:0000922	6	27	6.56E-03	Spindle pole
GO:0005794	20	176	7.63E-03	Golgi apparatus

	GO:0016020	17	122	2.03E-03	Membrane
	GO:0009986	16	132	8.82E-03	Cell surface
	GO:0005576	36	322	8.24E-04	Extracellular region
	GO:0016021	31	306	6.27E-03	Integral component of membrane
	GO:0030027	8	47	9.02E-03	Lamellipodium
	GO:0005615	33	327	5.35E-03	Extracellular space
	GO:0005789	14	109	8.58E-03	Endoplasmic reticulum membrane
	GO:0036020	2	2	4.62E-03	Endolysosome membrane
	GO:0030877	3	7	8.28E-03	Beta-catenin destruction complex
	GO:0036021	3	4	1.17E-03	Endolysosome lumen
MF	GO:0005524	65	537	5.79E-07	ATP binding
	GO:0004674	17	129	3.46E-03	Protein serine/threonine kinase activity
	GO:0009055	12	68	1.28E-03	Electron carrier activity
	GO:0004252	10	63	6.24E-03	Serine-type endopeptidase activity
	GO:0008201	9	55	7.53E-03	Heparin binding
	GO:0008144	6	28	7.79E-03	Drug binding
	GO:0005506	10	66	8.37E-03	Iron ion binding
	GO:0016887	4	13	8.06E-03	Atpase activity
	GO:0004222	7	34	5.28E-03	Metalloendopeptidase activity
	GO:0004716	4	8	1.12E-03	Receptor signaling protein tyrosine kinase activity
	GO:0004197	6	25	4.51E-03	Cysteine-type endopeptidase activity
	GO:0005149	2	2	4.62E-03	Interleukin-1 receptor binding
	GO:0005536	3	5	2.72E-03	Glucose binding
	GO:0043169	5	16	2.89E-03	Cation binding
	GO:0016812	2	2	4.62E-03	Hydrolase activity, acting on carbon-nitrogen (but not peptide) bonds, in cyclic amides
	GO:0008013	3	6	5.07E-03	Beta-catenin binding
	GO:0004029	3	6	5.07E-03	Aldehyde dehydrogenase (NAD) activity
	GO:0016303	3	4	1.17E-03	1-phosphatidylinositol-3-kinase activity
	GO:0015038	2	2	4.62E-03	Glutathione disulfide oxidoreductase

					activity
	GO:0046934	2	2	4.62E-03	Phosphatidylinositol-4,5-bisphosphate 3-kinase activity
BP	GO:0045087	26	200	5.07E-04	Innate immune response
	GO:0043066	32	212	7.23E-06	Negative regulation of apoptotic process
	GO:0006508	28	170	5.21E-06	Proteolysis
	GO:0006006	7	37	8.21E-03	Glucose metabolic process
	GO:0070207	4	8	1.12E-03	Protein homotrimerization
	GO:0044281	26	198	4.38E-04	Small molecule metabolic process
	GO:0007409	6	18	7.69E-04	Axonogenesis
	GO:0046777	13	94	6.34E-03	Protein autophosphorylation
	GO:0002250	4	11	4.28E-03	Adaptive immune response
	GO:0001816	4	9	1.88E-03	Cytokine production
	GO:0071222	8	29	4.27E-04	Cellular response to lipopolysaccharide
	GO:0045080	3	7	8.28E-03	Positive regulation of chemokine biosynthetic process
	GO:0042493	16	133	9.36E-03	Response to drug
	GO:0030168	15	111	4.52E-03	Platelet activation
	GO:0002576	9	55	7.53E-03	Platelet degranulation
	GO:0006935	5	21	9.49E-03	Chemotaxis
	GO:0071260	7	33	4.50E-03	Cellular response to mechanical stimulus
	GO:0045078	3	6	5.07E-03	Positive regulation of interferon-gamma biosynthetic process
	GO:0006096	8	25	1.42E-04	Glycolytic process
	GO:0005980	3	7	8.28E-03	Glycogen catabolic process
	GO:0046686	5	14	1.52E-03	Response to cadmium ion
	GO:0000281	3	7	8.28E-03	Mitotic cytokinesis
	GO:0007160	7	26	1.12E-03	Cell-matrix adhesion
	GO:0045780	3	7	8.28E-03	Positive regulation of bone resorption
	GO:0050435	3	5	2.72E-03	Beta-amyloid metabolic process
	GO:0007040	4	8	1.12E-03	Lysosome organization
	GO:0007494	2	2	4.62E-03	Midgut development

GO:0060444	4	8	1.12E-03	Branching involved in mammary gland duct morphogenesis
GO:0030316	4	11	4.28E-03	Osteoclast differentiation
GO:0033628	3	7	8.28E-03	Regulation of cell adhesion mediated by integrin
GO:0002437	3	7	8.28E-03	Inflammatory response to antigenic stimulus
GO:0032611	2	2	4.62E-03	Interleukin-1 beta production
GO:0042346	4	11	4.28E-03	Positive regulation of NF-kappab import into nucleus
GO:0003073	2	2	4.62E-03	Regulation of systemic arterial blood pressure
GO:0030207	3	5	2.72E-03	Chondroitin sulfate catabolic process
GO:0045356	2	2	4.62E-03	Positive regulation of interferon-alpha biosynthetic process
GO:0060740	3	6	5.07E-03	Prostate gland epithelium morphogenesis
GO:0042733	4	9	1.88E-03	Embryonic digit morphogenesis
GO:0061198	2	2	4.62E-03	Fungiform papilla formation
GO:0060789	3	4	1.17E-03	Hair follicle placode formation
GO:0010042	2	2	4.62E-03	Response to manganese ion
GO:0071372	3	5	2.72E-03	Cellular response to follicle-stimulating hormone stimulus
GO:0003420	2	2	4.62E-03	Regulation of growth plate cartilage chondrocyte proliferation
GO:0070527	3	4	1.17E-03	Platelet aggregation
GO:0014909	3	4	1.17E-03	Smooth muscle cell migration
GO:0042117	2	2	4.62E-03	Monocyte activation
GO:0010641	2	2	4.62E-03	Positive regulation of platelet-derived growth factor receptor signaling pathway
GO:0050927	3	5	2.72E-03	Positive regulation of positive chemotaxis
GO:0035116	4	11	4.28E-03	Embryonic hindlimb morphogenesis

GO:0032331	3	6	5.07E-03	Negative regulation of chondrocyte differentiation
GO:0050790	3	4	1.17E-03	Regulation of catalytic activity
GO:0051131	2	2	4.62E-03	Chaperone-mediated protein complex assembly
GO:0019953	2	2	4.62E-03	Sexual reproduction
GO:0055118	2	2	4.62E-03	Negative regulation of cardiac muscle contraction
GO:0048025	2	2	4.62E-03	Negative regulation of mrna splicing, via spliceosome
GO:0009966	3	4	1.17E-03	Regulation of signal transduction
GO:0061324	2	2	4.62E-03	Canonical Wnt signaling pathway involved in positive regulation of cardiac outflow tract cell proliferation
GO:0003344	2	2	4.62E-03	Pericardium morphogenesis

References

1. Chan, W.M. & Consortium, U. The UniProt Knowledgebase (UniProtKB): a freely accessible, comprehensive and expertly curated protein sequence database. *Genet Res* **92**, 78-79 (2010).
2. Kawashima, S. et al. AAindex: amino acid index database, progress report 2008. *Nucleic acids research* **36**, D202-205 (2008).
3. Holland, R.C. et al. BioJava: an open-source framework for bioinformatics. *Bioinformatics* **24**, 2096-2097 (2008).
4. Altschul, S.F. et al. Gapped BLAST and PSI-BLAST: a new generation of protein database search programs. *Nucleic acids research* **25**, 3389-3402 (1997).
5. Hubbard, S.J. & Thornton, J.M. Naccess. *Computer Program, Department of Biochemistry and Molecular Biology, University College London* **2** (1993).
6. Joosten, R.P. et al. A series of PDB related databases for everyday needs. *Nucleic acids research* **39**, D411-419 (2011).
7. Andersen, P., Nielsen, M. & Lund, O. Prediction of residues in discontinuous B-cell epitopes using protein 3D structures. *Protein science : a publication of the Protein Society* **15**, 2558-2567 (2006).
8. Mihel, J., Sikic, M., Tomic, S., Jeren, B. & Vlahovicek, K. PSAIA - protein structure and interaction analyzer. *BMC structural biology* **8**, 21 (2008).
9. Ren, J., Liu, Q., Ellis, J. & Li, J. Tertiary structure-based prediction of conformational B-cell epitopes through B factors. *Bioinformatics* **30**, i264-273 (2014).
